# Microscale Delivery Systems for Hydrophilic Active Ingredients in Functional Consumer Goods

**DOI:** 10.1002/wnan.70009

**Published:** 2025-04-13

**Authors:** Zhirui Guan, Daniele Baiocco, Andre Barros, Zhibing Zhang

**Affiliations:** ^1^ School of Chemical Engineering University of Birmingham Birmingham UK; ^2^ Healthcare Technology Institute, School of Chemical Engineering University of Birmingham Birmingham UK; ^3^ Procter & Gamble, Brussels Innovation Centre Strombeek‐Bever Belgium

**Keywords:** drug delivery, hydrophilic active ingredients, microcapsules, micromanipulation, microneedles, micropatches, microspheres, microsponges

## Abstract

Hydrophilic active ingredients play a crucial role in formulated consumer products, encompassing antioxidants, flavoring substances, and pharmaceuticals. Yet, their susceptibility to environmental factors, such as light, pH, temperature, and humidity, poses challenges to their stability and sustained release. Microencapsulation offers a promising avenue to address these challenges, facilitating stabilization, targeted delivery, and enhanced efficacy of hydrophilic actives. However, despite significant advancements in the field, microencapsulation of hydrophilic actives remains at the forefront of innovation. This is primarily due to the intrinsic characteristics of hydrophilic actives, including small molecular weight and thus high permeability through many microcarriers (e.g., shells), which often necessitate complex and costly technologies to be developed. Moreover, in light of escalating regulatory frameworks, the pursuit of biodegradable and other compliant materials suitable for the entrapment of hydrophilic ingredients is gaining momentum. These advancements aim to provide alternatives to currently used non‐degradable synthetic polymer materials. Research is currently pushing towards meeting these regulatory constraints via cutting‐edge technologies to engineer novel microscale delivery systems for hydrophilic active ingredients, including microcapsules, microspheres, microneedles, and micropatches. Although still in its infancy, this approach holds true potential for revolutionizing the future of formulated consumer goods.

## Graphical abstract

Understanding hydrophilic active ingredients by harnessing microscale delivery systems to enhance delivery, precision, efficacy, and shelf life of consumer goods, propelling us toward a sustainable circular economy.

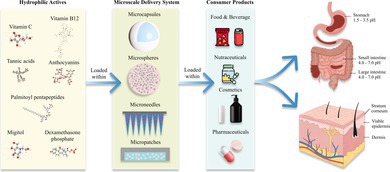

## 1 Introduction

Microscale delivery systems (MDS) can be used for segregating and safeguarding functional active ingredients (FAIs) within formulated products to control their release properties both in vitro and in vivo, in a broad range of industrial sectors, including health and personal care, cosmeceuticals, nutraceuticals, food, beverage, and agrochemicals (Arpicco et al. 2016; Chopra et al. 2022; Ciriminna and Pagliaro 2013; Mitragotri et al. 2014). Various techniques (e.g., interfacial/in situ polymerization, solvent evaporation, complex coacervation, micromolding) have been implemented to effectively produce MDS (Kim et al. 2019) and to encapsulate a plethora of FAIs, which can be lipophilic, hydrophilic, amphiphilic liquids, powders, haploid (fungi) or polyploid organisms (Casanova and Santos 2016; Jyothi et al. 2010; Lee et al. 2016; Windbergs et al. 2013; Yuan et al. 2024). Reported in the literature, hydrophilic FAIs that have been successfully isolated within different MDS encompass bioactive macromolecules, such as proteins (Mitragotri et al. 2014), polysaccharides (Sui et al. 2013), enzymes (Abdekhodaie et al. 2015; Keen et al. 2014) and smaller molecules, such as doxorubicin (Sui et al. 2013; Zhao, Luo, et al. 2015), polyphenols (Darniadi et al. 2019), carbamide peroxide (Bhaumik et al. 2015; Dogan et al. 2017), pigments, and artificial azo‐dyes (e.g., Allura red) (Sui et al. 2021). From vital nutrients like vitamin C (ascorbic acid) and B6 (pantothenic acid) to essential minerals, such as potassium chloride (KCl), the efficient delivery of hydrophilic FAIs has gained particular interest due to its potential for treating several health conditions, including scurvy, hypokalemia, and vitamin deficiencies of various aetiologies (Sui et al. 2021). However, the development of FAIs for such low molecular weight compounds has posed significant challenges. KCl was effectively entrapped within microspheres made of synthetic polymers, namely ethylcellulose (Wu et al. 2003b) and Eudragit (Wu et al. 2003a). However, these exhibited only a short‐term sustained release of KCl (6 h) in water, which may be insufficient for therapeutic applications. Alternatively, Sui et al. (2018) devised a methodology to fabricate KCl‐entrapping microcapsules for an extended sustained release (~2 days) in water, employing polystyrene sulfonate‐silica microspheres. Later, the same researchers developed microcapsules containing hydrophilic FAIs, both inorganic (KCl) and organic (Allura red), with outstanding barrier property. These showed no FAI leakage over a month in water, owing to the presence of two layers of melamine‐formaldehyde with a superhydrophobic ingredient (octadecyltrichlorosilane [OTS]) sandwiched in between, which marked a significant advancement in achieving prolonged sustained release of hydrophilic FAIs.

Notwithstanding, when compared to hydrophobic actives, the high solubilities of hydrophilic FAIs in water lead to easy leakage from microscale carriers into aqueous environments. To circumvent this, researchers have been actively working towards developing effective MDS. These systems aim to retain and stabilize hydrophilic FAIs over extended periods. Efforts have focused on engineering versatile, cost‐efficient, environmentally friendly systems capable of offering sustained release and improved efficacy in delivering hydrophilic FAIs to target sites (Hawthorne et al. 2022).

The selection of delivery systems including microcarrier materials is dictated by the specific properties of the FAIs and the intended application. This review centers on the latest advancements in MDS tailored for hydrophilic FAIs, including microcapsules, microspheres, micropatches, microsponges, and microneedles (MNs). Each system and key hydrophilic FAI are assessed for their unique advantages and suitability for specific applications, while being cognizant of the potential drawbacks, current challenges, and ambitions for the future.

## 2 Significance of MDS for Hydrophilic FAIs in Consumer Products

Implementation of MDS for hydrophilic FAIs holds significant importance in consumer products. Due to the presence of polar groups in their molecular backbone, hydrophilic FAIs often exhibit facilitated bioavailability and biomobility within the body fluids, compared to hydrophobic ones, especially for nutra‐ and pharmaceuticals (Arpicco et al. 2016). Popular hydrophilic FAIs in consumer goods include vitamin C (ascorbic acid) (Caritá et al. 2020) and vitamin B complexes (Santos et al. 2023), glycolic acid (Sharad 2013), caffeine (Bagheri et al. 2014), peptides, such as antioxidant glutathione (Liu et al. 2018), and herbal extracts like *Aloe vera* (Michalak 2023).

Each of these ingredients serves distinct purposes, from providing essential nutrients to offering skincare benefits or promoting overall wellness. However, hydrophilic active ingredients possess different physiochemical properties and sensitivities to environmental factors such as light, pH, oxygen, and temperature. Without proper protection, these ingredients may degrade or lose efficacy, compromising the quality and performance of the consumer products. Therefore, the implementation of suitable MDS becomes indispensable to ensure the efficient protection of these FAIs until they reach their intended site of action in a ‘metabolically’ active form (McClements 2015). By immobilizing these ingredients within appropriate carriers, such as microcapsules, microspheres or MNs, their stability can be significantly improved, extending their shelf life, and maintaining efficacy over time. Furthermore, the optimization of the overall efficiency of delivery of the actives allows for a reduction in loss of valuable ingredients that often require energy‐intensive processes to produce, leading to environmental benefits.

### 2.1 Key Hydrophilic FAIs With Commercial Interest

MDS facilitate controlled release and could ensure a sustained and consistent delivery of FAI to achieve desired therapeutic or cosmetic effects (Hardenia et al. 2019). The choice of specific MDS is tailored to the final application of the hydrophilic FAIs in consumer goods. For instance, skincare products may require delivery systems capable of penetrating the skin barrier to deliver active ingredients to deeper layers, whereas dietary supplements (e.g., vitamins) should rely on MDS capable of enhancing their absorption within the gastrointestinal tract (GIT) (Hoppel et al. 2015; Lee 2017).

#### 2.1.1 Foodstuff, Nutraceuticals, and Cosmeceuticals

Driven by increased self‐awareness and health consciousness of consumers, the market of nutraceuticals, fortified food, and beverages is burgeoning due to the increasing demand for products with additional health benefits (Chopra et al. 2022). Hydrophilic FAIs, including water‐soluble flavors, pigments, vitamins, herbal extracts, enzymes, minerals, and antioxidants, play a key role in these formulations (McClements 2015).

Vitamin C (L‐ascorbic acid) stands out for its multifaceted benefits in skincare, including collagen synthesis promotion, anti‐aging effects, and melanogenesis inhibition. However, its therapeutic potential is hampered by its notoriously poor stability. Vitamin C is prone to degradation under diverse conditions such as heat, light, and exposure to acidic or basic environments. Such instability poses significant challenges in formulating skincare products containing vitamin C, limiting its shelf life and efficacy (D'Ischia et al. 2015; Demiray et al. 2013; Hu et al. 2022).

While essential for red blood cell production and nervous system function, Vitamin B12 (cyanocobalamin) faces similar instability issues, including its high susceptibility to degradation when exposed to acid/alkaline environments, in addition to light and prolonged heating. These findings underscore the importance of careful considerations in formulated skincare products to preserve the integrity of vitamin B12 within appropriate MDS, ensuring its efficacy upon application (Chalella Mazzocato et al. 2019; Paniz et al. 2005).

Renowned for their antioxidant, anti‐inflammatory, antiphlogistic, and antiviral properties, polyphenols, such as phenolic acids and tannins, exhibit therapeutic potential in skin‐ and health‐care formulations. However, their instability presents a significant hurdle. Polyphenols are prone to degradation under various conditions, including pH fluctuations, temperature changes, and interactions with other formulation components. Consequently, maintaining the stability of polyphenols in skincare products remains a critical challenge for formulators seeking to harness their therapeutic benefits effectively (Deng et al. 2018; Kumar and Goel 2019).

Peptides, including palmitoyl pentapeptide (FOL‐005) play a vital role in skincare and cosmeceuticals, offering anti‐wrinkle, anti‐inflammatory, and hair growth stimulation properties. As with the other hydrophilic FAIs, their instability remains a concern. Peptides may undergo degradation when subjected to enzymatic activity, heat, pH, and temperature fluctuations (Mortazavi et al. 2019; Runnsjö et al. 2022). To tackle these challenges, MDS have been developed to segregate and protect hydrophilic FAIs, aiming to mask off‐tastes, improve their stability, bioavailability, and targeted delivery to specific regions, such as the small intestine and the colon (Lee 2017; Li et al. 2022). However, MDS may also face instability challenges; therefore, they must be skillfully designed to withstand specific environmental conditions.

#### 2.1.2 Pharmaceuticals

Various high‐demand pharmaceuticals are formulated using hydrophilic active ingredients, which are used in a broad spectrum of applications. However, many hydrophilic drugs for intramuscular and transdermal delivery, such as sumatriptan succinate, face challenges, including limited permeation and rapid skin irritation during delivery (Gomes et al. 2022; Kale and Momin 2014; Moeller and Jorgensen 2008).

For topical applications (e.g., creams, ointments), therapeutic molecules like Hydroxyzine HCl can be directly applied to the targeted site through the stratum corneum. However, permeability challenges, pH‐dependent instability due to the pH difference between the skin and the cream, and photosensitivity of the FAI significantly impair the effectiveness of the topical treatment. In addition, side effects (skin irritation, allergic reaction, erythema) often occur, exposing patients to potentially severe risks (Katare et al. 2010; Raza et al. 2014; Zaki Rizkalla et al. 2011).

For naso‐buccal and pulmonary applications, salbutamol sulfate is delivered via inhalers, nebulizers, and aerosols. However, its applicability faces hurdles associated with the short half‐life due to natural pulmonary clearance, enzymatic degradation, and rapid systemic absorption. This leads to low bioavailability at the targeted region, resulting in frequent dosing, low therapeutic efficiency, side effects, and subjecting patients to health risks (Knap et al. 2023; Kumar et al. 2023; Li et al. 2017).

Timolol Maleate is a nonselective beta blocker and ophthalmic medication to treat glaucoma, which has proven efficacious for most recipients. Notwithstanding, when administered topically, in the form of oil drops/gels, or systemically, adverse events have been documented, including the neuropsychiatric spectrum (Cimolai 2019), in addition to eye stinging and redness, conjunctivitis, and swollen eyelids (Abd‐Elal et al. 2020).

For proctological applications, cefuroxime sodium, a strong second‐generation antibiotic with bactericidal activity, can be delivered by suppositories, enemas, and rectal foams/ointments. However, when directly applied, it can cause severe rectal irritation, and its absorption may be interrupted by defecation (Jannin et al. 2014; Ofokansi and Adikwu 2007; Purohit et al. 2018; Xu et al. 2017).

In addition to the likelihood of side effects, when compared to hydrophobic drugs, the increased affinity of hydrophilic drugs to water enhances their initial mobility in body fluids but limits their ability to cross lipid barriers, leading to shorter drug lifetimes, lower bioavailability, and reduced absorption (Arpicco et al. 2016).

Pharmaceutical MDS are required to be intricately designed to address challenges associated with administering and delivering hydrophilic actives to specific treatment sites in the body (Adepu and Ramakrishna 2021). These systems aid in controlling drug dosage and mitigating potential treatment‐related side effects.

## 3 Microscale Delivery Systems

There are several types of MDS available for consumer goods, including microparticles (i.e., microspheres, microcapsules), micropatches, microsponges, and MNs (Baiocco, Lobel, et al. 2024; Banerjee et al. 2017; Choudhury et al. 2021; Huang et al. 2022; Singhvi et al. 2019). Optimizing MDS can be challenging, since it requires proper selection of the materials to form the microcarriers, which should be environmentally conscious and consumer friendly. Moreover, the manufacturing processes should be relatively inexpensive and preferably energy efficient (Lobel et al. 2024). With that being said, each of the MDS must guarantee the stability of actives during storage and excellent performance (controlled release) to the place of action, while masking potential off‐flavors and enhancing bioavailability. In this section, the key MDS are overviewed.

### 3.1 Microspheres

Microspheres are an important carrier for delivering hydrophilic active ingredients (FAIs) stored within homogeneous matrix‐based microcarriers. They are made from synthetic, natural, or bio‐inspired materials and vary in size from 1 μm to several hundred microns (Desai et al. 1996). This versatility allows easy integration into formulations like creams, gels, or liquid suspensions across diverse sectors such as food, beverages, pharmaceuticals, and advanced materials. Microspheres' high surface area‐to‐volume ratio enables rapid dissolution and absorption of hydrophilic compounds, boosting bioavailability and efficacy. Physiochemical properties like size, shape, chemical composition, and mechanical properties can be customized to suit specific delivery needs, for the sustained delivery of the actives within human body fluids or other environments (Lengyel et al. 2019; Mishra and Singh 2020; Runnsjö et al. 2022). For example, salbutamol sulfate can be entrapped within microspheres for treating specific pulmonary conditions. However, for inhalation administration, microparticle size should ideally range from 1 to 5 μm to avoid their deposition into the oropharynx or rapid clearance by exhalation. Hyaluronic acid (HA) microcarriers have been effective in enhancing pulmonary retention and therapeutic efficacy of salbutamol sulfate, owing to the strong biomuco‐adhesive properties of HA, while minimizing systemic exposure to the inhaled medicine (Knap et al. 2023; Li et al. 2017).

In intramuscular and transdermal delivery, microspheres sized approximately 2.5 μm have been engineered to immobilize hydrophilic substances like matrix‐2 protein virus‐like particles (M2e VLP). This approach not only prevented FAIs aggregation before administration but also enhanced their immunogenicity for mRNA vaccine delivery (Braz Gomes et al. 2021).

Shin et al. (2014) developed a novel method for preparing dopamine‐loaded polylactic‐co‐glycolic acid (PLGA)‐microspheres using double emulsion solvent evaporation. Strong intermolecular interactions between dopamine and the emulsifying polymer improved encapsulation efficiency. The addition of polyvinyl alcohol (PVA) in the inner water phase significantly enhanced loading efficiency from 1.6% ± 0.3% to 18.6% ± 9.2% (w/w) due to hydrogen bonding between dopamine and PVA, making these microspheres a potential candidate for Parkinson's disease treatment.

Chaisri et al. (2011) developed PLGA microspheres containing gentamicin sulfate (GS) by double emulsion solvent evaporation. It was found that increasing osmotic pressure in the external phase, coupled with higher PLGA concentrations, significantly enhanced the loading efficiency, achieving approximately 70%. These microspheres exhibited an initial jump release of GS upon exposure to aqueous environments, likely due to GS presence at their surface, followed by a slower, sustained release lasting up to 35 days. Subsequently, the release rate accelerated, possibly due to the incipient degradation of PLGA. The improved drug loading efficiency and prolonged release over 60 days suggest potential applications in various antibiotic treatments.

Overall, the selection and immobilization of hydrophilic FAIs within suitable matrices pose significant formulation challenges, necessitating careful consideration of delivery and therapeutic applications.

### 3.2 Microcapsules

Microcapsules stand out as one of the most promising MDS (Baiocco, Al‐Sharabi, et al. 2024; Baiocco et al. 2021b). The ideal microcapsule features a core‐shell configuration, consisting of a FAI (reservoir) enveloped within an organic, inorganic, or hybrid shell. Also, variants, such as irregularly shaped (e.g., elliptical) or multicore microcapsules, have been developed based on specific application requirements (Choudhury et al. 2021). Modulating the shell thickness allows for fine‐tuning of key properties like barrier and mechanical properties, crucial for overall microcapsule performance (Baiocco and Zhang 2022). Designing satisfactory microcapsules, particularly when encapsulating small hydrophilic FAIs, poses heavy challenges. This is due to various formulation parameters, such as core‐to‐shell mass ratio, interfacial energy, hygroscopicity/hydrophobicity, and thermal and rheological behaviors of core and shell materials, which are difficult to optimize and can affect the stability, functional, and performance properties of the formed microcapsule (Halahlah et al. 2023; Hategekimana et al. 2015; Ramakrishnan et al. 2018).

#### 3.2.1 Microencapsulation Techniques Relevant to Hydrophilic FAIs

Microencapsulation techniques encompass physical, chemical, and physico‐chemical methods. Physical methods utilize solid–liquid phase transitions or solvent evaporation to form a polymer‐based shell, employing techniques such as fluidized bed coating, spray chilling, and spray drying. Chemical methods rely on chemical reactions at the interface of emulsions and dispersions, involving polymerization of monomers through interfacial polymerization or in situ polymerization of small building blocks (monomers). Physicochemical methods involve shell formation through the precipitation of pre‐dissolved components onto the core surface, for example, coacervation, driven by temperature, pH variations, or increased ionic strength (Fu and Hu 2017). The morphology of microcapsules can be related to the morphology of the core. Specifically, liquid core microcapsules, featuring a single/composite shell, can be spherical as a result of mechanical forces around a spherical droplet under stirring (Fu and Hu 2017). Solvent evaporation often yields spherical microcapsules. However, the alignment of shell‐forming materials may also lead to microcapsules with elongated morphologies (e.g., eye‐shaped), as for complex coacervation (Baiocco et al. 2021a). Spray drying, solvent evaporation, and coacervation are elementary yet robust techniques that have long dominated the industrial scene (Deshmukh et al. 2016; Martins et al. 2013).

#### 3.2.2 Microcapsules Featuring Hydrophilic FAIs

Many hydrophilic FAIs are renowned for their nutritional and therapeutic benefits yet face challenges due to their chemical and thermal instability. Spray‐drying has emerged as a promising method to encapsulate hydrophilic compounds, enhancing their stability. For instance, Bagheri et al. (2014) developed caffeine‐loaded microcapsules with antioxidant nanoparticles, using alginate and calcium chloride as a crosslinker, resulting in structurally robust microcapsules (size 4–7.5 μm) with slow caffeine release for mitigating oxidative stress in the human body. Similarly, Darniadi et al. (2019) encapsulated anthocyanins and phenols from blueberries within a maltodextrin‐whey protein isolate matrix, achieving high encapsulation efficiency (75%–90%), offering potential for extending the shelf life of bioactive compounds in food and pharmaceutical products.

Trindade and Grosso (2000) utilized gum Arabic and rice starch as encapsulating materials for vitamin C, effectively enhancing its stability during storage. Similarly, Leyva‐López et al. (2019) achieved a 73.5% encapsulation efficiency of vitamin C by employing a blend of enzymatically modified starch and gum Arabic. In contrast, Hoyos‐Leyva et al. (2018) prepared spray‐dried microparticles loaded with vitamin C using taro starch spherical aggregates, yielding a lower encapsulation efficiency (~20.9%). Recently, Hu et al. (2022) employed chitosan and hydroxypropylmethylcellulose (HPMC) as gelling agents to fabricate microcapsules loaded with vitamin C. They employed a double emulsion (water (W_1_)‐oil (O)‐water (W_2_)) technique with sodium caseinate as the embedding material and hydrophilic emulsifier, followed by rapid spray‐drying. The resulting microcapsules (size ~6 μm) exhibited a high encapsulation efficiency (~92%) and promising stability likely attributed to the continuous chitosan coating. The interaction between the gel polymer and vitamin C could inhibit the leakage of vitamin C from W1 to W2, which represents an interesting strategy to stabilize the W_1_/O/W_2_ emulsion and enhance the encapsulation performance.

Silk fibroin (SF), a natural food‐grade biopolymer, demonstrates potential for addressing the instability of vitamin C. Liu et al. (2022) used spray‐drying to fabricate microcapsules (5 ± 3 μm) with tunable morphology and structure (e.g., hollow spongy, hollow smooth, hollow crumpled matrices). The mechanism behind this lies in the ability of SF's beta‐sheet structure to regulate shell hydrophobicity, influencing the stability and release properties of encapsulated vitamin C. This method accommodates high active ingredient concentrations, up to 50 wt%, offering versatility and environmentally conscious options for controlled release kinetics of vitamin C in formulated products. However, while this approach offers significant advantages, further investigation is warranted to optimize the encapsulation process and ensure long‐term stability and efficacy in practical skincare applications.

**FIGURE 1:**
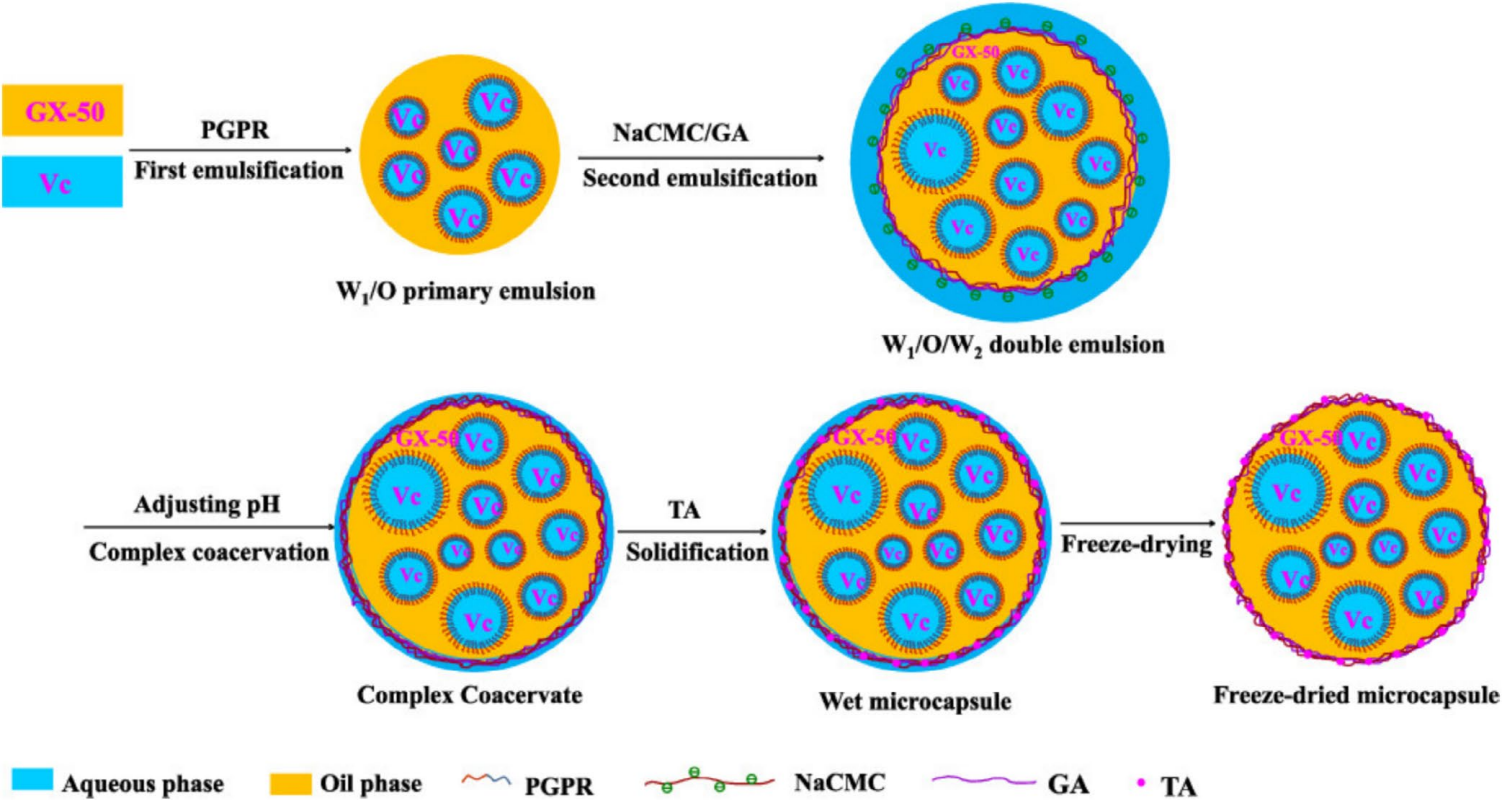
Schematic of the process for co‐encapsulating ascorbic acid (Vc) and GX‐50 (xanthoxylin) via double emulsification and complex coacervation of gum Arabic (GA) and carboxymethyl cellulose (NaCMC). Reproduced with permission from Wang et al. (2023). Copyright 2023 American Chemical Society.

Cheng et al. (2009) developed gelatine‐based microcapsules (size range: 5.0–44.1 μm) by emulsion hardening technique to achieve controlled release of vitamin C. Leveraging the moisture sensitivity of gelatine, the researchers aimed to deliver vitamin C, while anchoring the microcapsules onto cosmetic textiles, facilitating direct release onto the skin through textile‐skin contact. While no immediate cytotoxicity was observed on human cells, further studies are required to assess the efficacy of these microcapsules in real‐world cosmetic applications and their potential longer‐term effects on human skin. Comunian et al. (2013) used complex coacervation to encapsulate vitamin C within gelatine‐gum Arabic microcapsules ranging from 52 to 83 μm in size. They exhibited multiple nuclei and a spherical morphology, with remarkably high encapsulation efficiency (~98%), achieving enhanced stability of vitamin C compared to its free form. Lately, Wang et al. (2023), co‐encapsulated hydrophobic xanthoxylin (GX‐50) and vitamin C by a Water‐in‐Oil‐in‐Water (W_1_/O/W_2_) double emulsion–complex coacervation method, employing sodium carboxymethyl cellulose and gelatine. The encapsulation efficiencies for GX‐50 and vitamin C were ~86% and ~68%, respectively. Following oral administration, the typical mechanism of absorption of FAI across the intestinal epithelium is shown in Figure 1B.

Moreover, the study highlighted the capability of co‐encapsulating GX‐50 and vitamin C to penetrate through model skin membranes, suggesting potential applications in dermatological formulations or transdermal delivery systems. These findings underscore promising prospects for enhanced stability and delivery of active compounds in skincare and pharmaceutical formulations.

Other than spray drying and coacervation, interfacial polymerization was also applied to encapsulate vitamin C. Ripoll and Clement (2016) proposed a methodology involving 1,6‐hexamethylene‐diamine (HA) and terephthaloyl dichloride (TD) in the aqueous and the organic phase, respectively, which polymerized at their interface. The optimal formulation showed microcapsules (size 12.6–35.7 μm) with a high encapsulation efficiency of ~97%, and that only 33% of the encapsulated vitamin C was released over 1 month in water in dark conditions.

Moreover, interfacial polymerization was utilized for encapsulating water‐soluble herbicides, using chitosan and glutaraldehyde as a crosslinking agent (Yeom et al. 2000). Although the resulting microcapsules were promising, the use of glutaraldehyde is shrouded in controversy due to its toxicity, therefore limiting the potential application of such microcapsules. Takahashi et al. (2010) encapsulated hydrophilic solid powder (Bistetrazol diammonium) as a flame retardant using epoxy resin via interfacial polymerization. This method could achieve high encapsulation efficiency above 89%–100%, featuring payloads of 70%–85%. However, some epoxy resins are non‐biodegradable, which may be an issue for potential broader applications.

**FIGURE 2:**
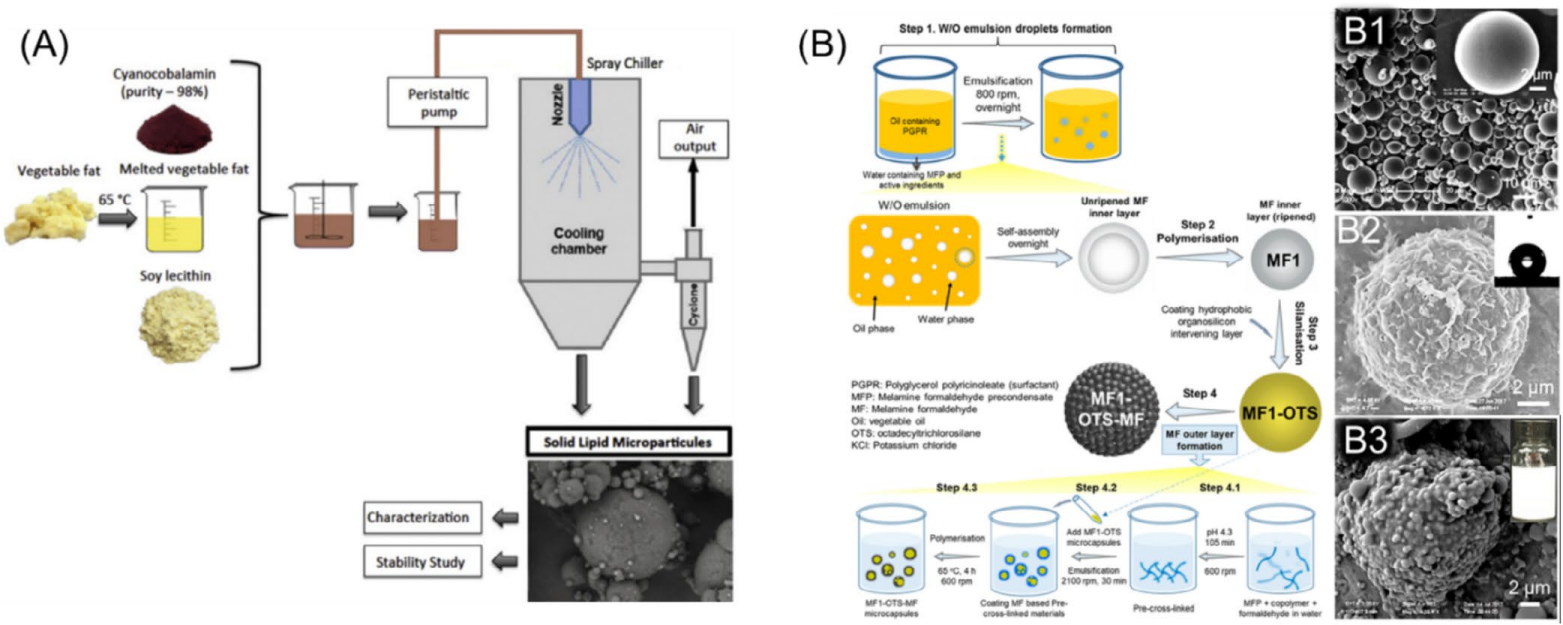
(A) Schematic of the fabrication and study of vitamin B12 microcapsules via spray‐chilling. Reprinted from Chalella Mazzocato et al. (2019); (B) Fabrication scheme of the MF1‐OTS‐MF microcapsules via two in situ polymerization steps and one dropwise coating step. The insets display: (B1) MF1; (B2) MF1‐OTS; (B3) MF1‐OTS‐MF2 microcapsules Sui et al. (2021). Both reprinted with permission. Elsevier copyright (2019 and 2020).

Differently, vitamin B12 (cyanocobalamin) was encapsulated by spray chilling (Figure 2A), using soy lecithin and vegetable fat (Chalella Mazzocato et al. 2019), with the intention to target the small intestine via oral delivery. Over 91% of vitamin B12 was preserved after 120 days under the condition of static storage at 25°C in darkness. However, a significant amount (60%–90%) of B12 was released to simulated gastric fluid, which is undesirable. Spray chilling was also employed for the fabrication of microcapsules laden with a proanthocyanidin‐rich cinnamon extract (PRCE), within solid lipid‐based shells. No significant degradation of PRCE was detected up to 90 days at 5°C, 25°C. Around 55% PRCE was released in the simulated gastric fluid after 180 min and almost kept the same level until 360 min in simulated intestinal fluid (Tulini et al. 2017). This demonstrates that the storage stability of PRCE was significantly improved after being encapsulated, but the shell of microcapsules could not fully prevent its premature release in the simulated gastric fluid. More research is required to improve the barrier properties of such microcapsules to ensure they have the right functionalities for a given application.

Lee et al. (2002) developed double‐walled microcapsules using a modified solvent evaporation technique for encapsulating etanidazole crystals. The microspheres consisted of an inner core of PLGA and an outer shell of poly(lactic acid) (PLLA). At a PLLA/PLGA mass ratio of 2:1, etanidazole solid crystal filaments were embedded in the PLGA core, achieving an encapsulation efficiency of ~57%. These microspheres exhibited controlled drug release, with low leakage (< 10%) in the first 6 days, indicating a real potential for prolonged and sustained release over 6–8 weeks. This possibly meets the duration requirements for antibiotic therapies to treat infections caused by protozoa (e.g., trichomoniasis, giardiasis, amebiasis).

**FIGURE 3:**
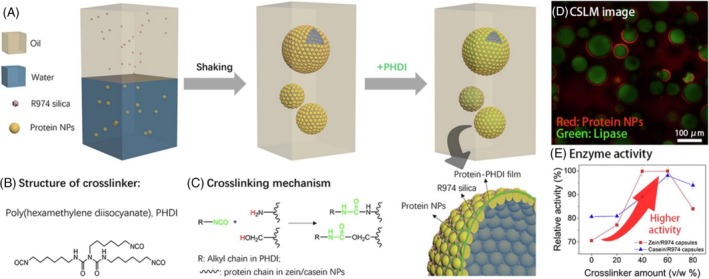
(A) Schematic of W/O Pickering emulsion templated enzyme encapsulation process and mechanisms utilizing zein/casein and polydiisocyanate (PHDI); (B) structure of crosslinker, PHDI; (C) crosslinking mechanism between functional groups in protein and isocyanate groups in PHDI; (D) CSLM image; (E) enzyme activity. Reprinted with permission from Liu et al. (2023). Elsevier copyright (2023).

In our recent study, Sui et al. (2021) utilized in situ polymerization to produce microcapsules featuring a dual‐shell made of melamine formaldehyde (MF) with a superhydrophobic OTS layer sandwiched between them (Figure 2B). These microcapsules, denoted as MF1‐OTS‐MF2, demonstrated a high encapsulation efficiency of 86%. The incorporation of the superhydrophobic OTS layer within the two synthetic layers effectively blocked the release of small hydrophilic actives, such as potassium ions (K^+^) and Allura Red dye, in aqueous environments for up to 1 month. Moreover, Liu et al. (2023) achieved Pickering emulsions (28–46 μm) with enhanced stability, armored by hydrophobic fumed silica nanoparticles, featuring zein‐casein networks crosslinked with polydiisocyanate, which demonstrated high resistance to de‐emulsification and water leakage over a week (Figure 3). Although these encapsulation approaches show promise for controlled release applications, the microcapsule shell is composed of non‐degradable material. The shifting regulatory landscape is encouraging further research into biodegradable alternatives, a trend which can be pursued within the field of hydrophilic AI encapsulation. Table 1 provides an overview of the applications of microcapsules containing hydrophilic FAIs based on their encapsulation method.

**TABLE 1:** Applications of microcapsules for hydrophilic FAIs based on their encapsulation method.

Method	FAI (hydrophilic)	Wall material	Size (μm)	Properties	Reference
Spray‐drying	Vitamin C	Sodium caseinate, Chitosan	6.0–6.4	Chitosan to reinforce the multilayer structure. Sustained release of Vitamin C.	Hu et al. (2022)
	Vitamin C	Silk fibroin (SF)	5 ± 3	Burst release of vitamin C controllable by SF beta‐sheet: higher beta‐sheet to enhance the hydrophobicity of the shell; biodegradability; controlled release.	Liu et al. (2022)
	Caffeine	Alginate	~4; (7.4 upon CaCl_2_ crosslinking)	Antioxidant activity; embedded in microparticle. Reinforced CaCl_2_‐crosslinked structure enabling slow controlled release of caffeine.	Bagheri et al. (2014)
	Anthocyanins and blueberry‐sourced polyphenols	Maltodextrin matrix with whey protein isolate (WPI)	—	Retention: 73% (polyphenols) and 95% (anthocyanins).	Darniadi et al. (2019)
Spray‐chilling	Vitamin B12 (cyanocobalamin)	Soy lecithin and vegetable fat	13.3–27.0	Static leakage: < 10% (over 91.1% protection of active after 120 days at 25°C in darkness). Slow diffusion of B12 (8%) in the beginning followed by constant release in simulated gastric fluids.	Chalella Mazzocato et al. (2019)
	Proanthocyanidin‐rich cinnamon extract (PRCE) and α‐tocopherol	Vegetable fat (hydrogenated soy and palm oils)	~80	PRCE stably stored in solid lipid microparticles up to 90 days under 5°C, 25°C, and 37°C. Gradual release for solid lipid microparticles in stimulated GI fluids.	Tulini et al. (2017)
Solvent evaporation	Etanidazole crystals	Double‐walled: PLGA (inner/core) and PLLA (outer/shell)	422.3 ± 174.5 432.3 ± 179.2	Encapsulation efficiency of 56.69% ± 0.21% at PLLA:PLGA (w/w) 2:1. In PBS buffer: 5% burst release (diffusion controlled). For normal sample 80% released over 10 days while irradiated sample achieved sustained release for more than 3 weeks. Biodegradability	Lee et al. (2002)
	Miglitol (W/O/W)	poly‐ε‐caprolactone (PCL)	54.7–140.0	Sustained release of MGL despite its shot half‐life. Biodegradability and biocompatibility.	Waghulde and Naik (2016)
Coacervation	Vitamin C (W/O)	Gelatine (crosslinked with formaldehyde)	5.0–44.1 (average: 24.6)	Controlled release of vitamin C onto the skin through textile contact.	Cheng et al. (2009)
Complex coacervation	Ascorbic acid (AA)/vitamin C	Gelatine and gum Arabic	51.6–83.8	High encapsulation efficiency ~98%. Stable, spherical, multinucleate microcapsules.	Comunian et al. (2013)
	Vitamin C and hydrophobic xanthoxylin (GX‐50)	Sodium carboxymethyl cellulose and gelatine crosslinked by Tannic acid	8.4–28.9	Encapsulation efficiency: GX‐50 (85.95%) and Vitamin C (67.35%). Co‐encapsulated GX‐50 and Vitamin C microcapsules able to penetrate through model skin membranes.	Wang et al. (2023)
Interfacial polymerization	BHT 2NH3 (powder)	Hydrazine (aqueous phase) Epoxy resin (organic phase)	—	High encapsulation efficiency (89%–100%) and high payload (70%–85%).	Takahashi et al. (2010)
	Vitamin C	1,6‐hexamethy‐lenediamine (HA) (aqueous phase) Terephtaloyl dichloride (TD) (organic phase)	12.6–35.7	High encapsulation efficiency (96.88%) and encapsulation yield (payload) of 67.41%. In darkness, 33% of the encapsulated vitamin C was released in 1 month in water.	Ripoll and Clement (2016)
In situ polymerization	KCl Allura red	Melamine formaldehyde (MF) with octadecyltrichloro‐silane (OTS)	78.3 (±0.3)	High encapsulation efficiency 85.8 ± 4.5. The sandwiched hydrophobic oil (OTS) layer prevented the release of K^+^ and Allura red in aqueous environment for 1 month.	Sui et al. (2021)
Crosslinked Pickering emulsion	Lipase enzyme	Hydrophobic fumed silica nanoparticles and zein, casein crosslinked by polydiisocyanate	28–46	High stability; no significant de‐emulsification or water leakage observed over a week.	Liu et al. (2023)

#### 3.2.3 Retained Hydrophilic FAIs: Comparison Between Microcapsules and Nanocapsules (NCs)

NCs differ from microcapsules primarily through their particle size, with NCs possessing diameters smaller than 1 μm and ideally falling within the range of 100–500 nm (Krieser et al. 2020; Morganti 2010). Table 2 presents a comparative analysis between microcapsules and NCs for the same hydrophilic active, focusing on parameters such as encapsulation efficiency (EE), yield, active retention, and delivery effects. Despite the smaller size of nanocarriers, their higher surface area‐to‐volume ratio often results in higher release rates (Hasani‐Sadrabadi et al. 2014). Indeed, NCs also have lower EE, as reported by Hassane Hamadou et al. (2020) who fabricated marine phospholipid‐based nanoliposomes (237–280 nm) loaded with vitamin C by a thin‐film evaporation method, yielding an EE of ~52%. Conversely, Ripoll and Clement (2016) produced 1,6‐hexamethylenediamine‐based microcapsules with an optimized EE of ~98%, which was almost two‐fold high as nanoliposomes. Similarly, anthocyanin‐loaded NCs (~133 nm) were fabricated by Wang, Tao, et al. (2021) using chondroitin sulfate and egg white protein via electrostatic interactions, achieving an EE of 42%–69%. In contrast, anthocyanin‐loaded microcapsules (21–48 μm) were produced by Mansour et al. (2020) via freeze‐drying employing soy protein isolate and gum Arabic, leading to an EE of ~93%–99%. Despite the lower EE, NCs appeared to enhance the permeability and spreadability of FAI across the body tissues, in addition to being better tolerated in intravenous drug administration (Maisha et al. 2021; Roberts et al. 2017; Singh and Nanda 2014).

**TABLE 2:** Applications of microcapsules and nanocapsules for hydrophilic FAIs based on their encapsulation method.

FAI	Scale	Encapsulation technique	Wall material	Size	Encapsulation efficiency	Retention	Target delivery	Reference
Vita‐min C	Micro	Interfacial polymerization	1,6‐hexamethylene‐diamine; terephtaloyl dichloride	12.6–35.7 μm	96.9%	33% of vitamin C released in 1 month stored in water avoiding light	—	Ripoll and Clement (2016)
	Micro	Complex coacervation	Sodium carboxymethyl cellulose and gelatine	8.4–28.9 μm	67.4%	—	Model skin membranes	Wang et al. (2023)
	Nano	Thin‐film evaporation	Marine phospholipids	236.7–279.7 nm	52.1%	Retention of 86.5% (EE: 52.09%) of vitamin C	—	Hassane Hamadou et al. (2020)
	Nano	Crosslinked Pickering emulsion	Chitosan‐phosphorylated cellulose nanocrystal	~460 nm	90.3% ± 0.4%	18% released after 14 days at pH 7; 27% released under GIT simulation after 8 h	Small intestine mucosa.	Baek et al. (2021)
Vita‐min B12	Micro	Spray‐chilling	Soy lecithin and vegetable fat	13.3–27.0 μm	76.6%–100.0%	91.1% protection of vitamin B12 after 120 days in darkness	Simulated gastric fluids.	Chalella Mazzocato et al. (2019)
	Nano	Double emulsion solvent evaporation	PLGA	190 nm	71%	Release (8 weeks): in aqueous suspension (i) 57.3% ± 2.5% (room temperature (RT)), (ii) 37.3% ± 7.3% (at 4°C); Dried form 13.2% ± 4.3% (RT) Simulated GIT: 9.5% ± 0.9% (2 h)	Gastrointestinal tract (GIT)	Ramalho et al. (2021)
Anthocyanin	Micro	Freeze‐drying	Soy protein isolate and gum Arabic	21.1 ± 2.7 μm	93.5%–98.8%	Retention: at $25^{{}^{\circ}}C$, 59.01% (30 days) and 39.4% (60 days); at $37^{{}^{\circ}}C$, 26.81% (30 days) and 17.3% (60 days).	GIT	Mansour et al. (2020)
	Nano	Electrostatic interactions	Chondroitin sulfate and egg white protein	133.2 ± 3.4 nm	42%–69%	—	—	Wang, Tao, et al. (2021)

### 3.3 MNs

**FIGURE 4:**
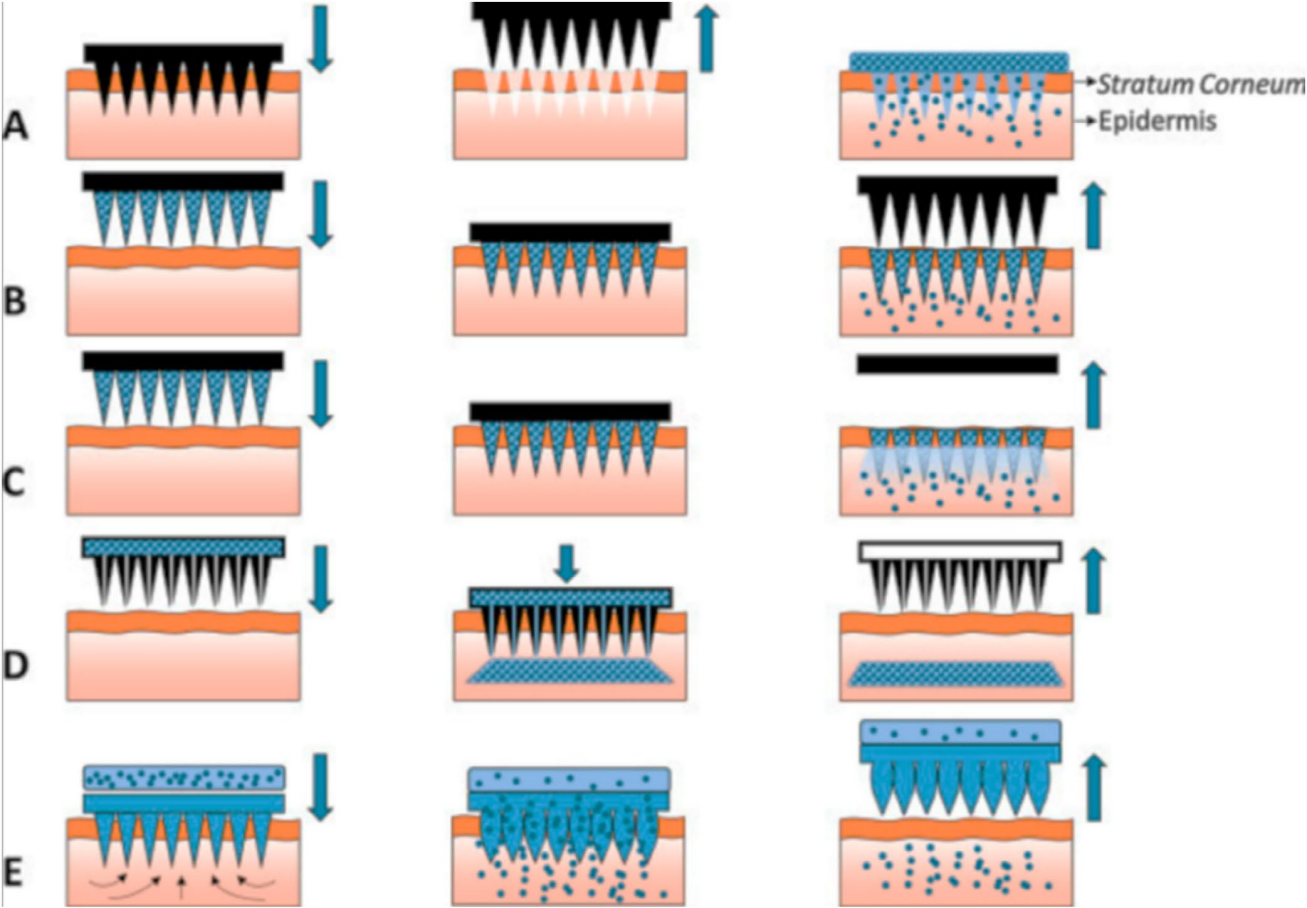
Illustration depicting the mechanisms of five distinct types of microneedles utilized for drug transdermal delivery: (A) Solid microneedles, (B) Coated microneedles, (C) Dissolvable microneedles, (D) Hollow microneedles, and (E) Hydrogel‐forming microneedles. Reprinted with permission from Larrañeta et al. (2016), CC BY 4.0.

MNs have emerged as a minimally invasive method for targeted delivery of hydrophilic compounds through the skin layers. These micron‐sized needles, often integrated into arrays, painlessly penetrate the stratum corneum, creating temporary microchannels that facilitate the transport of hydrophilic compounds to deeper skin layers (hypodermis). This enhances absorption and bioavailability across the dermal microcapillaries (Moffatt and Donnelly 2021). MNs can be divided into five types based on their mechanisms of action: (i) solid, porous, pocketed MNs (the drug is separated); (ii) dissolvable, biocompatible MNs (the drug is encased); (iii) hollow MNs (the drug is loaded as a reservoir in the back substrate); (iv) coated MNs (the drug is deposited at their outer surface); and (v) hydrogel‐forming MNs that can swell upon skin‐needling, sustainably releasing the drug (Aldawood et al. 2021; Huang et al. 2022; Ingrole et al. 2021) (Figure 4). Four categories of MNs are acknowledged based on their intended applications: (i) “poke‐and‐patch,” which involve microperforation followed by the application of a drug‐loaded patch; (ii) “coat‐and‐poke,” which entail the application of a formulation onto solid MNs; (iii) “poke‐and‐release,” consisting of soluble drug‐loaded MNs; and (iv) “poke‐and‐flow,” which are hollow, allowing for drug flow (Parhi 2018). Microneedle design is guided by four fundamental principles: drug loading, mechanical strength, geometric structure, and controlled release. Various materials, including metalloids (e.g., silicon and titanium) and soluble (bio)polymers like HA, are employed in MN fabrication (Parhi 2018). HA is a biodegradable macromolecule with excellent formability and drug loading capacity, yet it is mechanically weak. To enhance its mechanical strength, crosslinking and non‐crosslinking modification methods are used (Du, He, et al. 2021; Du, Zhang, et al. 2021; Huang et al. 2022; Luo et al. 2014). HA dissolvable MNs were also employed to load minoxidil for haircare applications. They were proven effective at enhancing the absorption rate through scalp skin, reducing the treatment period and associated side effects, as well as tackling alopecia in animals (Kim et al. 2022). Similarly, Lee et al. (2020) produced HA‐based MNs loaded with glutathione (GSH) for skin whitening and anti‐aging effect. In addition to masking off the undesirable smell of GSH, MNs enabled the permeation of the active, which was uniformly released over the required time period. Sumatriptan succinate was entrapped in dissolving polyvinylpyrrolidone‐based MNs for migraine treatment, exhibiting efficacious and precise administration (Ronnander et al. 2018). Mohammed and co‐workers showcased the enhanced transdermal delivery of cosmetic peptides on human skin by microneedling. Within MNs, melanostatin (a pseudo‐tripeptide), rigin (a tetrapeptide), and Pal‐KTTKS (a pentapeptide) inhibited melanin formation, reduced inflammation, and diminished wrinkles while improving skin complexion (Choi et al. 2014; Mohammed et al. 2014). Although the largest challenge in transdermal delivery remains to be the poor penetration through the stratum corneum (thickness ~10–40 μm), these MNs penetrated 304 ± 63 μm into the superficial dermis. This led to improved peptide delivery (Mohammed et al. 2014), reducing the risk of allergenic or infectious reactions (Huang et al. 2022).

### 3.4 Micropatches

Micropatches offer a convenient and non‐invasive method for delivering hydrophilic ingredients to the skin surface. These adhesive patches contain microscale reservoirs of active compounds, providing sustained release over extended periods and enabling targeted delivery to specific skin regions. Micropatches can overcome issues associated with needlephobic patients and improve therapeutic compliance (Fonte et al. 2013, 2015; Goldberg and Gomez‐Orellana 2003). They are versatile, accommodating various shapes and sizes, and can administer polypeptide hormone medications such as exenatide and calcitonin (Banerjee et al. 2017; Teutonico and Ponchel 2011). These micropatches were engineered to endure varying pH levels and mucoadhesive‐abundant environments, using synthetic cationic copolymer Eudragit E 100 based on dimethylaminoethyl methacrylate, as well as natural, swellable, gel‐forming biopolymers (pectin), to ensure the loaded drug is released within hours. Banerjee et al. (2017) investigated the feasibility of micropatches, measuring approximately 500 μm in diameter, which are capable of adhering to the intestinal mucosa of rodents, enhancing the delivery rate of insulin to achieve the rapid decrease of blood glucose level in vivo, improving therapeutic efficacy. Alternatively, Yamazoe et al. (2022) presented images of protein‐loaded micropatches of considerably smaller dimensions, approximately 118 μm in diameter, which exhibited effective adhesion to both small intestine and colon tissues of the rodents.

### 3.5 Microsponges

**FIGURE 5:**
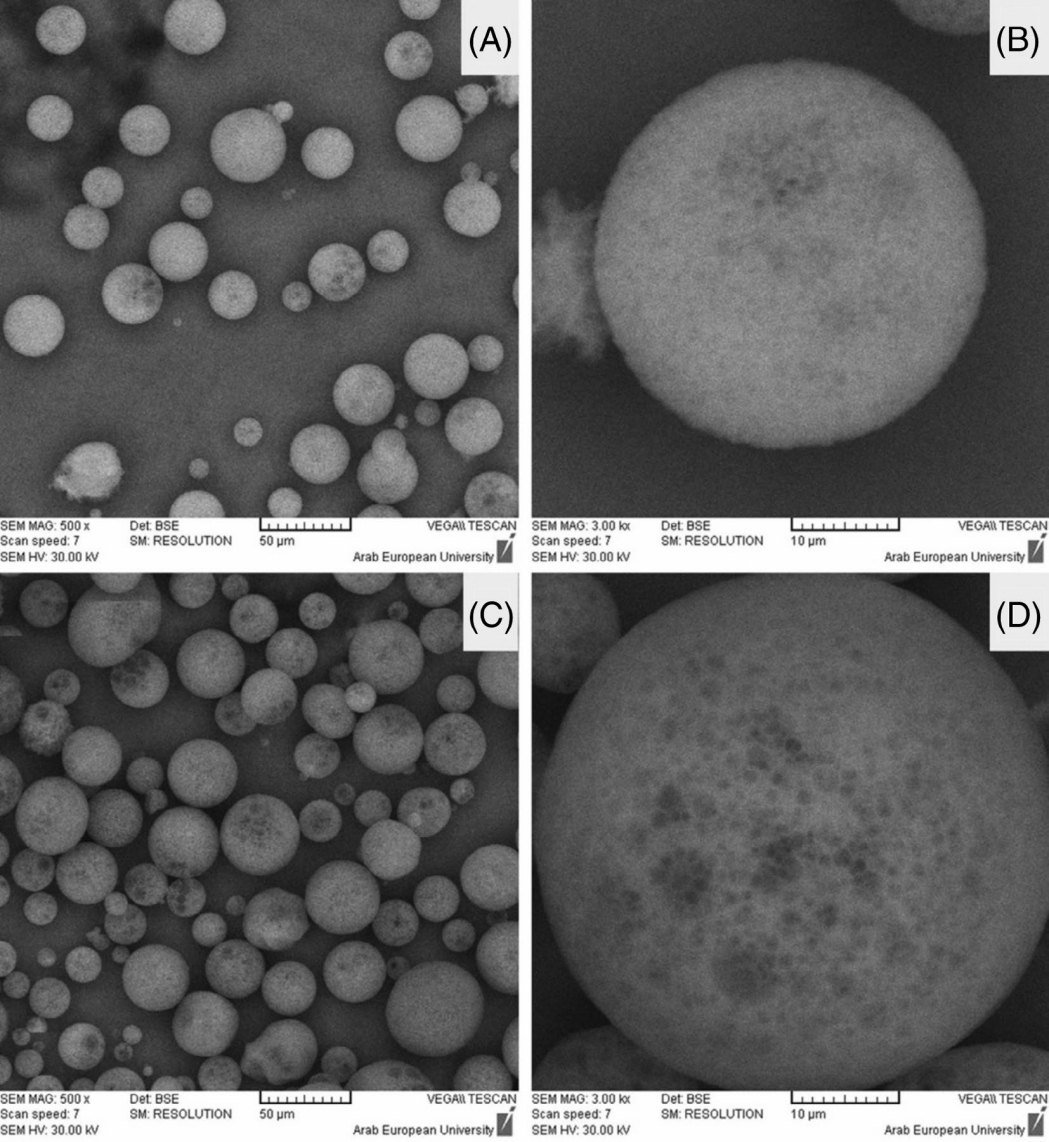
SEM images of clindamycin‐loaded microsponge (A) C5 formulation, 500×, (B) C5 formulation, 3000×, (C) C6 formulation, 500×, (D) C6 formulation, 3000×. Reprinted with permission from Khattab and Nattouf (2021), CC BY 4.0.

Microsponges represent another novel delivery system for the controlled delivery of hydrophilic FAIs. They typically range from 5 to 300 μm in size and possess a highly porous structure. Their small average pore size (0.25 μm) allows for topical release and subsequent systemic absorption of pharmaceutical and cosmeceutical actives while preventing the entry of larger microorganisms naturally found on human skin. Microsponges containing hydrophilic actives have found extensive application in many commercial formulations to treat several conditions, like acne, skin pigmentation, alopecia, sunburn, and hyperhidrosis (Mahant et al. 2020). For example, Lactrex moisturizing cream (SDR Pharmaceuticals Pvt. Ltd., India) utilizes lactic acid‐loaded microsponges, whereas oil‐free matte block sunscreen (Dermalogica LLC US) incorporates microsponges entrapping zinc gluconate. Additionally, Biophora Medical Skin Care (Ontario, Canada) enriches their skin‐exfoliation products with microsponges loaded with salicylic acid. Similarly, AMCOL Health and Beauty Solutions Inc. in the USA utilizes glycolic acid‐loaded microsponges as antiwrinkle and soothing agents in their sunscreen products (Mahant et al. 2020). Typical examples of microsponges are displayed in Figure 5.

Although they have proven stable in a broad pH range (1–11), with high active entrapment efficiencies (> 50%), microsponges are typically made of synthetic polymers (Li et al. 2013), which are often non‐biodegradable. Therefore, further research is required to provide alternative materials that meet regulatory requirements.

## 4 Challenges, Limitations, and Emerging Trends

### 4.1 Targeted Controlled Release of Hydrophilic FAIs and Mechanical Strength of MDS

FAIs are released from MDS by physical and chemical mechanisms (Petrusic and Koncar 2016). The former include the diffusion of embedded actives through the matrix/shell, which may also combine with the osmotic pressure effect and swelling/degradation of the shell materials, whereas the latter entail chemical/enzymatic activity to disrupt/degrade the active‐enveloping materials (Acharya and Park 2006; Petrusic and Koncar 2016).

Diffusion‐controlled release is the prevalent mechanism in both microcapsules and microspheres, where the active diffuses through pores or between polymer chains without dissolving/damaging the wall materials (Petrusic and Koncar 2016). Following encapsulation of water‐soluble diclofenac sodium (DS) in Eudragit RS100/RL100 microspheres, Deshmukh and Naik demonstrated diffusion‐driven sustained release of the active in vitro (Deshmukh and Naik 2013).

Osmotic systems rely on solvent ingress through a semipermeable wall, leading to solute diffusion of the active, which is then released by concentration gradient (Bruschi 2015).

Swellable systems involve hydrogel‐based materials swelling upon water contact, gradually releasing the pre‐loaded active, as described by Xia et al. (2019) in the case of hydrophilic tea polyphenol (TP) enveloped within a multilayer shell (i.e., outer layer: hydrophobic zein; middle layer: hybrid zein and gelatine proteins; inner layer: hydrophilic gelatine).

Chemically controlled mechanisms include erodible systems, where active release is activated by the hydrolytic/enzymatic degradation of wall materials (Peppas et al. 2000). Stimuli‐responsive microcapsules utilize smart‐responsive materials to react to environmental changes such as temperature, humidity, light, or pH (Petrusic and Koncar 2016).

In addition to evaluating the barrier properties and the associated release mechanisms, understanding the mechanical properties of MDS is pivotal, from their manufacturing to end‐use applications. Sui et al. demonstrated that microcapsules loaded with hydrophilic ingredients can be tested by micromanipulation, a technique developed by Prof. Zhibing Zhang's group at the University of Birmingham, UK (Sui et al. 2018; Zhang et al. 1999). This technique enables one to quantify important key mechanical property parameters of microcapsules, including their force versus displacement/deformation under compression, rupture force/tension, nominal rupture tension/stress, and displacement/deformation at rupture.

Interestingly, the same technique was applied to determine the mechanical response generated by individual MNs under compression (Du, Zhang, et al. 2021). Two types of HA MNs, with and without loaded model drugs, were tested by micromanipulation. The applied force in relation to the displacement of the MNs was measured, enabling the determination of rupture displacement and rupture force. The data points were utilized to construct a normal stress–deformation curve, to calculate the rupture stress and Young's modulus for each individual microneedle. Similar considerations have also been applied to microparticles (Baiocco et al. 2023).

### 4.2 Innovative Approaches for Immobilizing Hydrophilic FAIs

Electrohydrodynamic atomization (EHDA) is a promising method for fabricating micro‐ and nano‐sized particles (Arenas‐Jal et al. 2020; Smeets et al. 2017). It involves using a needle to eject a fluid, creating a mist of charged droplets under a high‐voltage electric field. Factors influencing EHDA include electrostatic force, electrical conductivity, needle gauge, distance to the collecting plate, and environmental conditions (Gu et al. 2013; Man et al. 2022). EHDA‐assisted microencapsulation can be modulated by the rapid solidification of microspheres and interactions between oppositely charged particles. Specifically, the electrosprayed droplets (core) are usually smaller and carry opposite charges to biopolymers (shell). Epigallocatechin gallate (EGCG), a hydrophilic bioactive compound, has been successfully encapsulated in zein and gelatine‐based microcapsules using coaxial electrospraying (Gómez‐Mascaraque et al. 2019). This coaxial electrospraying has shown improved antioxidant properties post in vitro digestion compared to uniaxial configuration, leading to a high encapsulation efficiency of α‐linolenic acid, between 65% and 90%.

Membrane emulsification is an advanced microencapsulation, known for its consistent control over droplet size (Maleki et al. 2021). In this process, the dispersed phase of an emulsion is pushed through membrane pores into a continuous phase, with droplets detaching at specific sizes or under shear force (Camelo‐Silva et al. 2022; Joscelyne and Trägårdh 2000). A continuous phase envelope is formed on the surface of the detaching monodisperse droplets, followed by the solidification of shell materials at the droplet interface (Christov et al. 2002). Catechol (CA) and DS have been successfully encapsulated in poly(vinyl alcohol) (PVA) microcapsules using membrane emulsification and chemical cross‐linking (Piacentini et al. 2020). Additionally, copper ions (hydrophilic) and α‐tocopherol (hydrophobic) were encapsulated within multicore poly‐caprolactone particles via azimuthally oscillating membrane emulsification, utilizing fish gelatine to stabilize internal water droplets, preventing coalescence. This method can lead to potential high throughput for industrial manufacturing, uniform emulsion, low shear force conditions, and encapsulation efficiencies up to 99% for copper ions and 93% for α‐tocopherol (Imbrogno et al. 2015). However, scaling up to industry production requires laborious and complex analysis, including many factors, such as cost effectiveness, process feasibility, and environmental impact assessment.

### 4.3 Stimuli‐Responsive Systems for Hydrophilic FAIs

Stimuli‐responsive MDS represent a cutting‐edge approach in controlled delivery, harnessing specific triggers (pH/temperature variations, light, humidity, enzymes, mechanical forces, ultrasound, redox potential, magnetism, and electricity) to precisely release FAIs at targeted sites (Lashgari et al. 2018; Li et al. 2015; Manzano and Vallet‐Regí 2019; Nucara et al. 2015; Peredo et al. 2021; Zhao, Liu, et al. 2015). Choi et al. (2021) designed thermo‐responsive microcapsules with a hydrogel membrane capable of selectively loading molecules with a hydrodynamic diameter smaller than the membrane cut‐off threshold (2–4 nm), such as water‐soluble fluorescein isothiocyanate‐dextran. These microcapsules exhibited temperature‐dependent changes in their hydrodynamic diameter and shell thickness due to positive/negative swelling with the temperature. Additionally, the inclusion of a photothermal agent, like polydopamine nanoparticles, within the microcapsule shell added extra functionalities, such as photo‐responsiveness.

Wang, Liu, et al. (2021) designed the temperature/pH dual‐stimuli‐responsive phase change microcapsules for smart drug delivery of hydrophilic anticancer doxorubicin hydrochloride (DOX). The researchers used n‐eicosane as the phase change material (PCM) as the core mixed with DOX and encapsulated with an acrylate‐functionalized silica shell by emulsion‐templated interfacial polycondensation. Subsequently, the pH‐responsive poly(*N*‐isopropylacrylamide‐co‐acrylic acid) layer was formed through surfactant‐assisted radical polymerization. The temperature/pH dual‐responsive microcapsules could release DOX controllably under acidic to neutral pH, exhibited a high thermal energy‐storage capacity of over 160 J/g, and achieved temperature regulation and drug delivery below 120°C.

Jeong et al. (2021) used triple emulsion droplets as templates to microfluidically prepare hydrogel microcapsules made of poly(ethylene glycol) diacrylate and poly‐vinyl alcohol, with a thin oil layer (hexane or FC‐70 with 2% Krytox‐PEG‐Krytox) for the entrapment of small hydrophilic actives, like erioglaucine and fluorescein sodium salt. The ensuing microcapsules exhibited long‐term retention up to 3 months, and the active could be released on demand upon mechanical compression (Jeong et al. 2021). Yang et al. (2020) presented a facile yet robust approach for fabricating pH‐responsive photonic crystal microcapsules via self‐assembly of block copolymers (polystyrene‐*b*‐poly(2‐vinylpyridine)). Rhodamine‐6G served as a model hydrophilic drug, which exhibited an accelerated release rate in acidic water, possibly due to swelling, accompanied by a red shift of its structural color. Such chromatic change offers a convenient means to monitor the release of hydrophilic FAI.

Overall, the encapsulation of hydrophilic FAIs offers an important yet complex route to programmable control their release through the required MDS, thereby enhancing the quality, performance, and shelf life of the final products. However, it is crucial to proceed with awareness to align with the evolving regulatory and techno‐economic criteria, prioritizing the use of biodegradable materials.

### 4.4 Safety and Regulatory Concerns

Regulation (EU) 2023/2055 restricts the use of “synthetic polymer microparticles” either singularly or in mixtures, wherein synthetic polymers constitute at least 1% of their weight or exhibit a continuous surface coating of the polymer, with at least 1% of the particles equal to or smaller than 5 mm in size. Conversely, products made with inorganic materials (not containing carbon), natural polymers, biodegradable polymers, or those soluble in water remain permissible.

By 2029, the synthetic polymer content in certain formulations may not exceed 0.01%. Synthetic polymers in the scope of the legislation are commonly used to encapsulate active ingredients and aid in the deposition of such actives, which enables consumer benefits and a more efficient use of precious ingredients. To maintain this consumer and environmental benefit post 2029, the regulation necessitates innovation, that is, development of biodegradable polymer alternatives in MDS and to foster the exploration of novel materials.

## 5 Conclusion and Outlook

Hydrophilic FAIs, encompassing vitamins, peptides, proteins, and polyphenols, play pivotal roles in numerous modern consumer products. They exhibit diverse functionalities such as antioxidative and anti‐inflammatory properties and provide essential nutrients and coveted cosmetic benefits, alongside medical and therapeutic effects.

Despite their significance, many hydrophilic FAIs are sensitive to environmental factors and thus face hurdles in storage and controlled delivery due to the limited choice of materials in alignment with regulatory considerations. Effectively delivering these FAIs to their intended points of action in end‐use applications presents a formidable challenge.

MDS have emerged as a promising solution for preserving and delivering hydrophilic ingredients, particularly those targeting release within the gastrointestinal tract or transdermal delivery to the hypodermic microcapillaries through the stratum corneum.

While microcapsules, microspheres, MNs, micropatches, and microsponges have garnered considerable attention in the field, the state‐of‐the‐art MDS still confront challenges. Although various wall materials and manufacturing methods have been explored to achieve high encapsulation efficiency and improve delivery efficacy with controlled release, issues persist regarding the retention time and stability of hydrophilic actives within the MDS during storage and application, representing critical research bottlenecks.

Furthermore, the evolving landscape of consumer demands and evolving regulatory landscapes regarding environmental impacts presents new challenges for the development of MDS. Addressing these challenges necessitates further substantial research of novel systems, utilizing regulatory compliant materials only, which reflect a critical consideration for the future of FAI delivery technologies.

## Author Contributions

**Zhirui Guan:** conceptualization (supporting), formal analysis (lead), investigation (lead), methodology (lead), validation (equal), visualization (equal), writing – original draft (lead). **Daniele Baiocco:** conceptualization (supporting), formal analysis (supporting), investigation (supporting), methodology (supporting), supervision (supporting), validation (supporting), visualization (supporting), writing – review and editing (supporting). **Andre Barros:** conceptualization (supporting), formal analysis (supporting), funding acquisition (supporting), investigation (supporting), methodology (supporting), project administration (supporting), resources (supporting), supervision (supporting), validation (supporting), visualization (supporting), writing – review and editing (supporting). **Zhibing Zhang:** conceptualization (lead), formal analysis (supporting), funding acquisition (lead), investigation (supporting), methodology (supporting), project administration (lead), resources (lead), supervision (lead), writing – review and editing (lead).

## Conflicts of Interest

The authors declare no conflicts of interest.

## Related WIREs Articles

Protein cargo encapsulation by virus‐like particles: Strategies and applications

## Data Availability

Data sharing is not applicable to this article as no new data were created or analyzed in this study.

## References

[wnan70009-bib-0001] Abdekhodaie, M. J. , J. Cheng , and X. Y. Wu . 2015. “Effect of Formulation Factors on the Bioactivity of Glucose Oxidase Encapsulated Chitosan–Alginate Microspheres: In Vitro Investigation and Mathematical Model Prediction.” Chemical Engineering Science 125: 4–12. 10.1016/j.ces.2014.11.010.

[wnan70009-bib-0002] Abd‐Elal, R. M. A. , G. H. Elosaily , S. Gad , E.‐S. Khafagy , and Y. Mostafa . 2020. “Full Factorial Design, Optimization, In Vitro and Ex Vivo Studies of Ocular Timolol‐Loaded Microsponges.” Journal of Pharmaceutical Innovation 15, no. 4: 651–663. 10.1007/s12247-019-09418-z.

[wnan70009-bib-0003] Acharya, G. , and K. Park . 2006. “Mechanisms of Controlled Drug Release From Drug‐Eluting Stents.” Advanced Drug Delivery Reviews 58, no. 3: 387–401. 10.1016/j.addr.2006.01.016. 16546289

[wnan70009-bib-0004] Adepu, S. , and S. Ramakrishna . 2021. “Controlled Drug Delivery Systems: Current Status and Future Directions.” Molecules 26, no. 19: 5905. https://www.mdpi.com/1420‐3049/26/19/5905. 34641447 10.3390/molecules26195905 PMC8512302

[wnan70009-bib-0005] Aldawood, F. K. , A. Andar , and S. Desai . 2021. “A Comprehensive Review of Microneedles: Types, Materials, Processes, Characterizations and Applications.” Polymers 13, no. 16: 2815. https://www.mdpi.com/2073‐4360/13/16/2815. 34451353 10.3390/polym13162815 PMC8400269

[wnan70009-bib-0006] Arenas‐Jal, M. , J. M. Suñé‐Negre , and E. García‐Montoya . 2020. “An Overview of Microencapsulation in the Food Industry: Opportunities, Challenges, and Innovations.” European Food Research and Technology 246, no. 7: 1371–1382. 10.1007/s00217-020-03496-x.

[wnan70009-bib-0007] Arpicco, S. , L. Battaglia , P. Brusa , et al. 2016. “Recent Studies on the Delivery of Hydrophilic Drugs in Nanoparticulate Systems.” Journal of Drug Delivery Science and Technology 32: 298–312. 10.1016/j.jddst.2015.09.004.

[wnan70009-bib-0008] Baek, J. , M. Ramasamy , N. C. Willis , D. S. Kim , W. A. Anderson , and K. C. Tam . 2021. “Encapsulation and Controlled Release of Vitamin C in Modified Cellulose Nanocrystal/Chitosan Nanocapsules.” Current Research in Food Science 4: 215–223. 10.1016/j.crfs.2021.03.010. 33937869 PMC8076697

[wnan70009-bib-0009] Bagheri, L. , A. Madadlou , M. Yarmand , and M. E. Mousavi . 2014. “Spray‐Dried Alginate Microparticles Carrying Caffeine‐Loaded and Potentially Bioactive Nanoparticles.” Food Research International 62: 1113–1119. 10.1016/j.foodres.2014.05.040.

[wnan70009-bib-0010] Baiocco, D. , M. Al‐Sharabi , B. T. Lobel , O. J. Cayre , A. F. Routh , and Z. Zhang . 2024. “Eco‐Friendly Fungal Chitosan‐Silica Dual‐Shell Microcapsules With Tailored Mechanical and Barrier Properties for Potential Consumer Product Applications.” ACS Omega 9: 28385–28396. 10.1021/acsomega.4c02287. 38973847 PMC11223154

[wnan70009-bib-0011] Baiocco, D. , B. T. Lobel , M. Al‐Sharabi , O. J. Cayre , A. F. Routh , and Z. Zhang . 2024. “Environmentally Friendly Calcium Carbonate‐Polydopamine Microcapsules With Superior Mechanical, Barrier, and Adhesive Properties.” Sustainable Materials and Technologies 41: e01001. 10.1016/j.susmat.2024.e01001.

[wnan70009-bib-0012] Baiocco, D. , J. A. Preece , and Z. Zhang . 2021a. “Encapsulation of Hexylsalicylate in an Animal‐Free Chitosan‐Gum Arabic Shell by Complex Coacervation.” Colloids and Surfaces A: Physicochemical and Engineering Aspects 625: 126861. 10.1016/j.colsurfa.2021.126861.

[wnan70009-bib-0013] Baiocco, D. , J. A. Preece , and Z. Zhang . 2021b. “Microcapsules With a Fungal Chitosan‐Gum Arabic‐Maltodextrin Shell to Encapsulate Health‐Beneficial Peppermint Oil.” Food Hydrocolloids for Health 1: 100016. 10.1016/j.fhfh.2021.100016.

[wnan70009-bib-0014] Baiocco, D. , and Z. Zhang . 2022. “Microplastic‐Free Microcapsules to Encapsulate Health‐Promoting Limonene Oil.” Molecules 27, no. 21: 7215. 10.3390/molecules27217215. 36364038 PMC9659182

[wnan70009-bib-0015] Baiocco, D. , Z. Zhang , Y. He , and Z. Zhang . 2023. “Relationship Between the Young's Moduli of Whole Microcapsules and Their Shell Material Established by Micromanipulation Measurements Based on Diametric Compression Between Two Parallel Surfaces and Numerical Modelling.” Micromachines 14, no. 1: 123. https://www.mdpi.com/2072‐666X/14/1/123. 36677184 10.3390/mi14010123 PMC9867421

[wnan70009-bib-0016] Banerjee, A. , J. Wong , R. Gogoi , T. Brown , and S. Mitragotri . 2017. “Intestinal Micropatches for Oral Insulin Delivery.” Journal of Drug Targeting 25, no. 7: 608–615. 10.1080/1061186X.2017.1300664. 28266884

[wnan70009-bib-0017] Bhaumik, J. , N. S. Thakur , P. K. Aili , A. Ghanghoriya , A. K. Mittal , and U. C. Banerjee . 2015. “Bioinspired Nanotheranostic Agents: Synthesis, Surface Functionalization, and Antioxidant Potential.” ACS Biomaterials Science & Engineering 1, no. 6: 382–392. 10.1021/ab500171a. 33445243

[wnan70009-bib-0018] Braz Gomes, K. , S. D'Sa , G. L. Allotey‐Babington , S.‐M. Kang , and M. J. D'Souza . 2021. “Transdermal Vaccination With the Matrix‐2 Protein Virus‐Like Particle (M2e VLP) Induces Immunity in Mice Against Influenza A Virus.” Vaccine 9, no. 11: 1324. https://www.mdpi.com/2076‐393X/9/11/1324. 10.3390/vaccines9111324 PMC8619166 34835255

[wnan70009-bib-0019] Bruschi, M. L. 2015. “4 ‐ Main Mechanisms to Control the Drug Release.” In Strategies to Modify the Drug Release From Pharmaceutical Systems, edited by M. L. Bruschi , 37–62. Woodhead Publishing. https://www.sciencedirect.com/science/article/pii/B9780081000922000047.

[wnan70009-bib-0020] Camelo‐Silva, C. , S. Verruck , A. Ambrosi , and M. Di Luccio . 2022. “Innovation and Trends in Probiotic Microencapsulation by Emulsification Techniques.” Food Engineering Reviews 14, no. 3: 462–490. 10.1007/s12393-022-09315-1.

[wnan70009-bib-0021] Caritá, A. C. , B. Fonseca‐Santos , J. D. Shultz , B. Michniak‐Kohn , M. Chorilli , and G. R. Leonardi . 2020. “Vitamin C: One Compound, Several Uses. Advances for Delivery, Efficiency and Stability.” Nanomedicine: Nanotechnology, Biology and Medicine 24: 102117. 10.1016/j.nano.2019.102117. 31676375

[wnan70009-bib-0022] Casanova, F. , and L. Santos . 2016. “Encapsulation of Cosmetic Active Ingredients for Topical Application—A Review.” Journal of Microencapsulation 33, no. 1: 1–17. 10.3109/02652048.2015.1115900. 26612271

[wnan70009-bib-0023] Chaisri, W. , A. H. Ghassemi , W. E. Hennink , and S. Okonogi . 2011. “Enhanced Gentamicin Loading and Release of PLGA and PLHMGA Microspheres by Varying the Formulation Parameters.” Colloids and Surfaces B: Biointerfaces 84, no. 2: 508–514. 10.1016/j.colsurfb.2011.02.006. 21353499

[wnan70009-bib-0024] Chalella Mazzocato, M. , M. Thomazini , and C. S. Favaro‐Trindade . 2019. “Improving Stability of Vitamin B12 (Cyanocobalamin) Using Microencapsulation by Spray Chilling Technique.” Food Research International 126: 108663. 10.1016/j.foodres.2019.108663. 31732070

[wnan70009-bib-0025] Cheng, S. Y. , M. C. W. Yuen , C. W. Kan , K. K. L. Cheuk , C. H. Chui , and K. H. Lam . 2009. “Cosmetic Textiles With Biological Benefits: Gelatin Microcapsules Containing Vitamin C.” International Journal of Molecular Medicine 24, no. 4: 411–419. 10.3892/ijmm_00000247. 19724879

[wnan70009-bib-0026] Choi, Y. H. , J.‐S. Hwang , S. H. Han , C.‐S. Lee , S.‐J. Jeon , and S.‐H. Kim . 2021. “Thermo‐Responsive Microcapsules With Tunable Molecular Permeability for Controlled Encapsulation and Release.” Advanced Functional Materials 31, no. 24: 2100782. 10.1002/adfm.202100782.

[wnan70009-bib-0027] Choi, Y. L. , E. J. Park , E. Kim , D. H. Na , and Y.‐H. Shin . 2014. “Dermal Stability and in Vitro Skin Permeation of Collagen Pentapeptides (KTTKS and Palmitoyl‐KTTKS).” Biomolecules & Therapeutics 22, no. 4: 321–327. 10.4062/biomolther.2014.053. 25143811 PMC4131521

[wnan70009-bib-0028] Chopra, A. S. , R. Lordan , O. K. Horbańczuk , et al. 2022. “The Current Use and Evolving Landscape of Nutraceuticals.” Pharmacological Research 175: 106001. 10.1016/j.phrs.2021.106001. 34826602

[wnan70009-bib-0029] Choudhury, N. , M. Meghwal , and K. Das . 2021. “Microencapsulation: An Overview on Concepts, Methods, Properties and Applications in Foods.” Food Frontiers 2, no. 4: 426–442. 10.1002/fft2.94.

[wnan70009-bib-0030] Christov, N. C. , D. N. Ganchev , N. D. Vassileva , N. D. Denkov , K. D. Danov , and P. A. Kralchevsky . 2002. “Capillary Mechanisms in Membrane Emulsification: Oil‐in‐Water Emulsions Stabilized by Tween 20 and Milk Proteins.” Colloids and Surfaces A: Physicochemical and Engineering Aspects 209, no. 1: 83–104. 10.1016/S0927-7757(02)00167-X.

[wnan70009-bib-0031] Cimolai, N. 2019. “Neuropsychiatric Adverse Events From Topical Ophthalmic Timolol.” Clinical Medicine & Research 17, no. 3–4: 90–96. 10.3121/cmr.2019.1486. 31462538 PMC6886891

[wnan70009-bib-0032] Ciriminna, R. , and M. Pagliaro . 2013. “Sol–Gel Microencapsulation of Odorants and Flavors: Opening the Route to Sustainable Fragrances and Aromas.” Chemical Society Reviews 42, no. 24: 9243–9250. 10.1039/C3CS60286A. 24077399

[wnan70009-bib-0033] Comunian, T. A. , M. Thomazini , A. J. G. Alves , F. E. de Matos Junior , J. C. de Carvalho Balieiro , and C. S. Favaro‐Trindade . 2013. “Microencapsulation of Ascorbic Acid by Complex Coacervation: Protection and Controlled Release.” Food Research International 52, no. 1: 373–379. 10.1016/j.foodres.2013.03.028.

[wnan70009-bib-0034] Darniadi, S. , I. Ifie , P. Ho , and B. S. Murray . 2019. “Evaluation of Total Monomeric Anthocyanin, Total Phenolic Content and Individual Anthocyanins of Foam‐Mat Freeze‐Dried and Spray‐Dried Blueberry Powder.” Journal of Food Measurement and Characterization 13, no. 2: 1599–1606. 10.1007/s11694-019-00076-w.

[wnan70009-bib-0155] Desai, M. P. , V. Labhasetwar , and G. L. Amidon , and R. J. Levy . 1996. “Gastrointestinal Uptake of Biodegradable Microparticles: Effect of Particle Size.” Pharmaceutical Research 13, no. 12: 1838–1845. 10.1023/A:1016085108889. 8987081

[wnan70009-bib-0035] Demiray, E. , Y. Tulek , and Y. Yilmaz . 2013. “Degradation Kinetics of Lycopene, β‐Carotene and Ascorbic Acid in Tomatoes During Hot Air Drying.” LWT—Food Science and Technology 50, no. 1: 172–176. 10.1016/j.lwt.2012.06.001.

[wnan70009-bib-0036] Deng, J. , H. Yang , E. Capanoglu , H. Cao , and J. Xiao . 2018. “9—Technological Aspects and Stability of Polyphenols.” In Polyphenols: Properties, Recovery, and Applications, edited by C. M. Galanakis , 295–323. Woodhead Publishing. https://www.sciencedirect.com/science/article/pii/B9780128135723000099.

[wnan70009-bib-0037] Deshmukh, R. , P. Wagh , and J. Naik . 2016. “Solvent Evaporation and Spray Drying Technique for micro‐ and Nanospheres/Particles Preparation: A Review.” Drying Technology 34, no. 15: 1758–1772. 10.1080/07373937.2016.1232271.

[wnan70009-bib-0038] Deshmukh, R. K. , and J. B. Naik . 2013. “Diclofenac Sodium‐Loaded Eudragit Microspheres: Optimization Using Statistical Experimental Design.” Journal of Pharmaceutical Innovation 8, no. 4: 276–287. 10.1007/s12247-013-9167-9.

[wnan70009-bib-0039] D'Ischia, M. , K. Wakamatsu , F. Cicoira , et al. 2015. “Melanins and Melanogenesis: From Pigment Cells to Human Health and Technological Applications.” Pigment Cell & Melanoma Research 28, no. 5: 520–544. 10.1111/pcmr.12393. 26176788

[wnan70009-bib-0040] Dogan, E. M. , F. Sudur Zalluhoglu , and N. Orbey . 2017. “Effect of Potassium Ion on the Stability and Release Rate of Hydrogen Peroxide Encapsulated in Silica Hydrogels.” AICHE Journal 63, no. 2: 409–417. 10.1002/aic.15406.

[wnan70009-bib-0041] Du, G. , P. He , J. Zhao , et al. 2021. “Polymeric Microneedle‐Mediated Transdermal Delivery of Melittin for Rheumatoid Arthritis Treatment.” Journal of Controlled Release 336: 537–548. 10.1016/j.jconrel.2021.07.005. 34237400

[wnan70009-bib-0042] Du, G. , Z. Zhang , P. He , Z. Zhang , and X. Sun . 2021. “Determination of the Mechanical Properties of Polymeric Microneedles by Micromanipulation.” Journal of the Mechanical Behavior of Biomedical Materials 117: 104384. 10.1016/j.jmbbm.2021.104384. 33592344

[wnan70009-bib-0043] Fonte, P. , F. Araújo , S. Reis , and B. Sarmento . 2013. “Oral Insulin Delivery: How Far Are We?” Journal of Diabetes Science and Technology 7, no. 2: 520–531. 10.1177/193229681300700228. 23567010 PMC3737653

[wnan70009-bib-0044] Fonte, P. , F. Araújo , C. Silva , et al. 2015. “Polymer‐Based Nanoparticles for Oral Insulin Delivery: Revisited Approaches.” Biotechnology Advances 33, no. 6 Pt 3: 1342–1354. 10.1016/j.biotechadv.2015.02.010. 25728065

[wnan70009-bib-0045] Fu, F. , and L. Hu . 2017. “15—Temperature Sensitive Colour‐Changed Composites.” In Advanced High Strength Natural Fibre Composites in Construction, edited by M. Fan and F. Fu , 405–423. Woodhead Publishing. https://www.sciencedirect.com/science/article/abs/pii/B9780081004111000157.

[wnan70009-bib-0046] Goldberg, M. , and I. Gomez‐Orellana . 2003. “Challenges for the Oral Delivery of Macromolecules.” Nature Reviews. Drug Discovery 2, no. 4: 289–295. 10.1038/nrd1067. 12669028

[wnan70009-bib-0047] Gomes, K. B. , I. Menon , P. Bagwe , L. Bajaj , S.‐M. Kang , and M. J. D'Souza . 2022. “Enhanced Immunogenicity of an Influenza Ectodomain Matrix‐2 Protein Virus‐Like Particle (M2e VLP) Using Polymeric Microparticles for Vaccine Delivery.” Viruses 14, no. 9: 1920. 10.3390/v14091920. 36146733 PMC9506217

[wnan70009-bib-0048] Gómez‐Mascaraque, L. G. , F. Tordera , M. J. Fabra , M. Martínez‐Sanz , and A. Lopez‐Rubio . 2019. “Coaxial Electrospraying of Biopolymers as a Strategy to Improve Protection of Bioactive Food Ingredients.” Innovative Food Science & Emerging Technologies 51: 2–11. 10.1016/j.ifset.2018.03.023.

[wnan70009-bib-0049] Gu, Z. , A. A. Aimetti , Q. Wang , et al. 2013. “Injectable Nano‐Network for Glucose‐Mediated Insulin Delivery.” ACS Nano 7, no. 5: 4194–4201. 10.1021/nn400630x. 23638642 PMC4107450

[wnan70009-bib-0050] Halahlah, A. , V. Piironen , K. S. Mikkonen , and T. M. Ho . 2023. “Polysaccharides as Wall Materials in Spray‐Dried Microencapsulation of Bioactive Compounds: Physicochemical Properties and Characterization.” Critical Reviews in Food Science and Nutrition 63, no. 24: 6983–7015. 10.1080/10408398.2022.2038080. 35213281

[wnan70009-bib-0051] Hardenia, A. , N. Maheshwari , S. S. Hardenia , S. K. Dwivedi , R. Maheshwari , and R. K. Tekade . 2019. “Chapter 1—Scientific Rationale for Designing Controlled Drug Delivery Systems.” In Basic Fundamentals of Drug Delivery, edited by R. K. Tekade , 1–28. Academic Press. 10.1016/B978-0-12-817909-3.00001-7.

[wnan70009-bib-0052] Hasani‐Sadrabadi, M. M. , V. Karimkhani , F. S. Majedi , et al. 2014. “Microfluidic‐Assisted Self‐Assembly of Complex Dendritic Polyethylene Drug Delivery Nanocapsules.” Advanced Materials 26, no. 19: 3118–3123. 10.1002/adma.201305753. 24610685

[wnan70009-bib-0053] Hassane Hamadou, A. , W.‐C. Huang , C. Xue , and X. Mao . 2020. “Formulation of Vitamin C Encapsulation in Marine Phospholipids Nanoliposomes: Characterization and Stability Evaluation During Long Term Storage.” LWT 127: 109439. 10.1016/j.lwt.2020.109439.

[wnan70009-bib-0054] Hategekimana, J. , K. G. Masamba , J. Ma , and F. Zhong . 2015. “Encapsulation of Vitamin E: Effect of Physicochemical Properties of Wall Material on Retention and Stability.” Carbohydrate Polymers 124: 172–179. 10.1016/j.carbpol.2015.01.060. 25839808

[wnan70009-bib-0055] Hawthorne, D. , A. Pannala , S. Sandeman , and A. Lloyd . 2022. “Sustained and Targeted Delivery of Hydrophilic Drug Compounds: A Review of Existing and Novel Technologies From Bench to Bedside.” Journal of Drug Delivery Science and Technology 78: 103936. 10.1016/j.jddst.2022.103936.

[wnan70009-bib-0056] Hoppel, M. , G. Reznicek , H. Kählig , H. Kotisch , G. P. Resch , and C. Valenta . 2015. “Topical Delivery of Acetyl Hexapeptide‐8 From Different Emulsions: Influence of Emulsion Composition and Internal Structure.” European Journal of Pharmaceutical Sciences 68: 27–35. 10.1016/j.ejps.2014.12.006. 25497319

[wnan70009-bib-0057] Hoyos‐Leyva, J. D. , A. Chavez‐Salazar , F. Castellanos‐Galeano , L. A. Bello‐Perez , and J. Alvarez‐Ramirez . 2018. “Physical and Chemical Stability of l‐Ascorbic Acid Microencapsulated Into Taro Starch Spherical Aggregates by Spray Drying.” Food Hydrocolloids 83: 143–152. 10.1016/j.foodhyd.2018.05.002.

[wnan70009-bib-0058] Hu, S. , Z. Ding , G. Zhang , et al. 2022. “Fabrication and Spray‐Drying Microencapsulation of Vitamin C‐Loaded W1/O/W2 Emulsions: Influence of Gel Polymers in the Internal Water Phase on Encapsulation Efficiency, Reconstituted Stability, and Controlled Release Properties.” LWT 170: 114113. 10.1016/j.lwt.2022.114113.

[wnan70009-bib-0059] Huang, Y. , H. Yu , L. Wang , et al. 2022. “Research Progress on Cosmetic Microneedle Systems: Preparation, Property and Application.” European Polymer Journal 163: 110942. 10.1016/j.eurpolymj.2021.110942.

[wnan70009-bib-0060] Imbrogno, A. , M. M. Dragosavac , E. Piacentini , G. T. Vladisavljević , R. G. Holdich , and L. Giorno . 2015. “Polycaprolactone Multicore‐Matrix Particle for the Simultaneous Encapsulation of Hydrophilic and Hydrophobic Compounds Produced by Membrane Emulsification and Solvent Diffusion Processes.” Colloids and Surfaces B: Biointerfaces 135: 116–125. 10.1016/j.colsurfb.2015.06.071. 26241923

[wnan70009-bib-0061] Ingrole, R. S. J. , E. Azizoglu , M. Dul , J. C. Birchall , H. S. Gill , and M. R. Prausnitz . 2021. “Trends of Microneedle Technology in the Scientific Literature, Patents, Clinical Trials and Internet Activity.” Biomaterials 267: 120491. 10.1016/j.biomaterials.2020.120491. 33217629 PMC8042615

[wnan70009-bib-0062] Jannin, V. , G. Lemagnen , P. Gueroult , D. Larrouture , and C. Tuleu . 2014. “Rectal Route in the 21st Century to Treat Children.” Advanced Drug Delivery Reviews 73: 34–49. 10.1016/j.addr.2014.05.012. 24871671

[wnan70009-bib-0063] Jeong, H.‐S. , E. Kim , C. Nam , et al. 2021. “Hydrogel Microcapsules With a Thin Oil Layer: Smart Triggered Release via Diverse Stimuli.” Advanced Functional Materials 31, no. 18: 2009553. 10.1002/adfm.202009553.

[wnan70009-bib-0064] Joscelyne, S. M. , and G. Trägårdh . 2000. “Membrane Emulsification—A Literature Review.” Journal of Membrane Science 169, no. 1: 107–117. 10.1016/S0376-7388(99)00334-8.

[wnan70009-bib-0065] Jyothi, N. V. N. , P. M. Prasanna , S. N. Sakarkar , K. S. Prabha , P. S. Ramaiah , and G. Y. Srawan . 2010. “Microencapsulation Techniques, Factors Influencing Encapsulation Efficiency.” Journal of Microencapsulation 27, no. 3: 187–197. 10.3109/02652040903131301. 20406093

[wnan70009-bib-0066] Kale, T. R. , and M. Momin . 2014. “Needle Free Injection Technology—An Overview.” Innovations in Pharmacy 5, no. 1: 148. 10.24926/iip.v5i1.330.

[wnan70009-bib-0067] Katare, O. , K. Raza , B. Singh , and S. Dogra . 2010. “Novel Drug Delivery Systems in Topical Treatment of Psoriasis: Rigors and Vigors.” Indian Journal of Dermatology, Venereology, and Leprology 76, no. 6: 612–621. 10.4103/0378-6323.72451. 21079304

[wnan70009-bib-0068] Keen, P. H. R. , N. K. H. Slater , and A. F. Routh . 2014. “Encapsulation of Amylase in Colloidosomes.” Langmuir 30, no. 8: 1939–1948. 10.1021/la4047897. 24517717

[wnan70009-bib-0069] Khattab, A. , and A. Nattouf . 2021. “Optimization of Entrapment Efficiency and Release of Clindamycin in Microsponge Based Gel.” Scientific Reports 11, no. 1: 23345. 10.1038/s41598-021-02826-7. 34857863 PMC8639917

[wnan70009-bib-0070] Kim, M. J. , K.‐Y. Seong , D. S. Kim , et al. 2022. “Minoxidil‐Loaded Hyaluronic Acid Dissolving Microneedles to Alleviate Hair Loss in an Alopecia Animal Model.” Acta Biomaterialia 143: 189–202. 10.1016/j.actbio.2022.02.011. 35202857

[wnan70009-bib-0071] Kim, Y. , S. A. Bhattaccharjee , M. Beck‐Broichsitter , and A. K. Banga . 2019. “Fabrication and Characterization of Hyaluronic Acid Microneedlesto Enhance Delivery of Magnesium Ascorbyl Phosphate Into Skin.” Biomedical Microdevices 21, no. 4: 104. 10.1007/s10544-019-0455-0. 31773286

[wnan70009-bib-0072] Knap, K. , K. Kwiecień , K. Reczyńska‐Kolman , and E. Pamuła . 2023. “Inhalable Microparticles as Drug Delivery Systems to the Lungs in a Dry Powder Formulations.” Regenerative Biomaterials 10: rbac099. 10.1093/rb/rbac099. 36683752 PMC9845529

[wnan70009-bib-0073] Krieser, K. , J. Emanuelli , R. M. Daudt , et al. 2020. “Taste‐Masked Nanoparticles Containing Saquinavir for Pediatric Oral Administration.” Materials Science and Engineering: C 117: 111315. 10.1016/j.msec.2020.111315. 32919675

[wnan70009-bib-0074] Kumar, M. , A. R. Hilles , S. H. Almurisi , A. Bhatia , and S. Mahmood . 2023. “Micro and Nano‐Carriers‐Based Pulmonary Drug Delivery System: Their Current Updates, Challenges, and Limitations—A Review.” JCIS Open 12: 100095. 10.1016/j.jciso.2023.100095.

[wnan70009-bib-0075] Kumar, N. , and N. Goel . 2019. “Phenolic Acids: Natural Versatile Molecules With Promising Therapeutic Applications.” Biotechnology Reports 24: e00370. 10.1016/j.btre.2019.e00370. 31516850 PMC6734135

[wnan70009-bib-0076] Larrañeta, E. , R. E. M. Lutton , A. D. Woolfson , and R. F. Donnelly . 2016. “Microneedle Arrays as Transdermal and Intradermal Drug Delivery Systems: Materials Science, Manufacture and Commercial Development.” Materials Science and Engineering: R: Reports 104: 1–32. 10.1016/j.mser.2016.03.001.

[wnan70009-bib-0077] Lashgari, S. , A. R. Mahdavian , H. Arabi , V. Ambrogi , and V. Marturano . 2018. “Preparation of Acrylic PCM Microcapsules With Dual Responsivity to Temperature and Magnetic Field Changes.” European Polymer Journal 101: 18–28. 10.1016/j.eurpolymj.2018.02.011.

[wnan70009-bib-0078] Lee, S. 2017. “Chapter 4—Strategic Design of Delivery Systems for Nutraceuticals.” In Nanotechnology Applications in Food, edited by A. E. Oprea and A. M. Grumezescu , 65–86. Academic Press. https://www.sciencedirect.com/science/article/abs/pii/B9780128119426000042.

[wnan70009-bib-0079] Lee, T. H. , J. Wang , and C.‐H. Wang . 2002. “Double‐Walled Microspheres for the Sustained Release of a Highly Water Soluble Drug: Characterization and Irradiation Studies.” Journal of Controlled Release 83, no. 3: 437–452. 10.1016/S0168-3659(02)00235-3. 12387951

[wnan70009-bib-0080] Lee, T. Y. , T. M. Choi , T. S. Shim , R. A. M. Frijns , and S.‐H. Kim . 2016. “Microfluidic Production of Multiple Emulsions and Functional Microcapsules.” Lab on a Chip 16, no. 18: 3415–3440. 10.1039/C6LC00809G. 27470590

[wnan70009-bib-0081] Lee, Y. , S. Kumar , S. H. Kim , et al. 2020. “Odorless Glutathione Microneedle Patches for Skin Whitening.” Pharmaceutics 12, no. 2: 100. 10.3390/pharmaceutics12020100. 32012667 PMC7076458

[wnan70009-bib-0082] Lengyel, M. , N. Kállai‐Szabó , V. Antal , A. J. Laki , and I. Antal . 2019. “Microparticles, Microspheres, and Microcapsules for Advanced Drug Delivery.” Scientia Pharmaceutica 87, no. 3: 20. 10.3390/scipharm87030020.

[wnan70009-bib-0083] Leyva‐López, R. , H. M. Palma‐Rodríguez , A. López‐Torres , J. Capataz‐Tafur , L. A. Bello‐Pérez , and A. Vargas‐Torres . 2019. “Use of Enzymatically Modified Starch in the Microencapsulation of Ascorbic Acid: Microcapsule Characterization, Release Behavior and In Vitro Digestion.” Food Hydrocolloids 96: 259–266. 10.1016/j.foodhyd.2019.04.056.

[wnan70009-bib-0084] Li, H. , H. Jiang , M. Zhao , Y. Fu , and X. Sun . 2015. “Intracellular Redox Potential‐Responsive Micelles Based on Polyethylenimine‐Cystamine‐Poly(ε‐Caprolactone) Block Copolymer for Enhanced miR‐34a Delivery.” Polymer Chemistry 6, no. 11: 1952–1960. 10.1039/C4PY01623H.

[wnan70009-bib-0085] Li, M. , X. He , R. Zhao , Q. Shi , Y. Nian , and B. Hu . 2022. “Hydrogels as Promising Carriers for the Delivery of Food Bioactive Ingredients.” Frontiers in Nutrition 9: 1006520. 10.3389/fnut.2022.1006520. 36238460 PMC9551458

[wnan70009-bib-0086] Li, S. S. , G. F. Li , L. Liu , et al. 2013. “Evaluation of Paeonol Skin‐Target Delivery From Its Microsponge Formulation: In Vitro Skin Permeation and In Vivo Microdialysis.” PLoS One 8, no. 11: e79881. 10.1371/journal.pone.0079881. 24278204 PMC3835837

[wnan70009-bib-0087] Li, Y. , M. Han , T. Liu , D. Cun , L. Fang , and M. Yang . 2017. “Inhaled Hyaluronic Acid Microparticles Extended Pulmonary Retention and Suppressed Systemic Exposure of a Short‐Acting Bronchodilator.” Carbohydrate Polymers 172: 197–204. 10.1016/j.carbpol.2017.05.020. 28606525

[wnan70009-bib-0088] Liu, L. , J. Wei , K. M. Ho , K. Y. Chiu , and T. Ngai . 2023. “Capsules Templated From Water‐In‐Oil Pickering Emulsions for Enzyme Encapsulation.” Journal of Colloid and Interface Science 629: 559–568. 10.1016/j.jcis.2022.09.106. 36179576

[wnan70009-bib-0089] Liu, M. , P.‐E. Millard , H. Urch , et al. 2022. “Microencapsulation of High‐Content Actives Using Biodegradable Silk Materials.” Small 18, no. 31: 2201487. 10.1002/smll.202201487. 35802906

[wnan70009-bib-0090] Liu, Q. , C.‐h. Deng , and N. Sun . 2018. “Hydrophilic Tripeptide‐Functionalized Magnetic Metal–Organic Frameworks for the Highly Efficient Enrichment of N‐Linked Glycopeptides.” Nanoscale 10, no. 25: 12149–12155. 10.1039/C8NR03174F. 29920571

[wnan70009-bib-0091] Lobel, B. T. , D. Baiocco , M. Al‐Sharabi , A. F. Routh , Z. Zhang , and O. J. Cayre . 2024. “Current Challenges in Microcapsule Designs and Microencapsulation Processes: A Review.” ACS Applied Materials & Interfaces 16, no. 31: 40326–40355. 10.1021/acsami.4c02462. 39042830 PMC11311140

[wnan70009-bib-0092] Luo, C. , J. Zhao , M. Tu , R. Zeng , and J. Rong . 2014. “Hyaluronan Microgel as a Potential Carrier for Protein Sustained Delivery by Tailoring the Crosslink Network.” Materials Science & Engineering. C, Materials for Biological Applications 36, no. 1: 301–308. 10.1016/j.msec.2013.12.021. 24433916

[wnan70009-bib-0093] Mahant, S. , S. Kumar , S. Nanda , and R. Rao . 2020. “Microsponges for Dermatological Applications: Perspectives and Challenges.” Asian Journal of Pharmaceutical Sciences 15, no. 3: 273–291. 10.1016/j.ajps.2019.05.004. 32636947 PMC7327759

[wnan70009-bib-0094] Maisha, N. , M. Rubenstein , C. J. Bieberich , and E. Lavik . 2021. “Getting to the Core of It All: Nanocapsules to Mitigate Infusion Reactions Can Promote Hemostasis and be a Platform for Intravenous Therapies.” Nano Letters 21, no. 21: 9069–9076. 10.1021/acs.nanolett.1c02746. 34714087

[wnan70009-bib-0095] Maleki, M. , C. de Loubens , K. Xie , E. Talansier , H. Bodiguel , and M. Leonetti . 2021. “Membrane Emulsification for the Production of Suspensions of Uniform Microcapsules With Tunable Mechanical Properties.” Chemical Engineering Science 237: 116567. 10.1016/j.ces.2021.116567.

[wnan70009-bib-0096] Man, Y. , C. Zhou , B. Adhikari , Y. Wang , T. Xu , and B. Wang . 2022. “High Voltage Electrohydrodynamic Atomization of Bovine Lactoferrin and Its Encapsulation Behaviors in Sodium Alginate.” Journal of Food Engineering 317: 110842. 10.1016/j.jfoodeng.2021.110842.

[wnan70009-bib-0097] Mansour, M. , M. Salah , and X. Xu . 2020. “Effect of Microencapsulation Using Soy Protein Isolate and Gum Arabic as Wall Material on Red Raspberry Anthocyanin Stability, Characterization, and Simulated Gastrointestinal Conditions.” Ultrasonics Sonochemistry 63: 104927. 10.1016/j.ultsonch.2019.104927. 31952001

[wnan70009-bib-0098] Manzano, M. , and M. Vallet‐Regí . 2019. “Ultrasound Responsive Mesoporous Silica Nanoparticles for Biomedical Applications.” Chemical Communications 55, no. 19: 2731–2740. 10.1039/C8CC09389J. 30694270 PMC6667338

[wnan70009-bib-0099] Martins, R. M. , S. V. Pereira , S. Siqueira , W. F. Salomão , and L. A. P. Freitas . 2013. “Curcuminoid Content and Antioxidant Activity in Spray Dried Microparticles Containing Turmeric Extract.” Food Research International 50, no. 2: 657–663. 10.1016/j.foodres.2011.06.030.

[wnan70009-bib-0100] McClements, D. J. 2015. “Encapsulation, Protection, and Release of Hydrophilic Active Components: Potential and Limitations of Colloidal Delivery Systems.” Advances in Colloid and Interface Science 219: 27–53. 10.1016/j.cis.2015.02.002. 25747522

[wnan70009-bib-0101] Michalak, M. 2023. “Plant Extracts as Skin Care and Therapeutic Agents.” International Journal of Molecular Sciences 24, no. 20: 15444. 10.3390/ijms242015444. 37895122 PMC10607442

[wnan70009-bib-0102] Mishra, B. , and J. Singh . 2020. “Chapter 4—Novel Drug Delivery Systems and Significance in Respiratory Diseases.” In Targeting Chronic Inflammatory Lung Diseases Using Advanced Drug Delivery Systems, edited by K. Dua , P. M. Hansbro , R. Wadhwa , M. Haghi , L. G. Pont , and K. A. Williams , 57–95. Academic Press. https://www.sciencedirect.com/science/article/pii/B9780128206584000042.

[wnan70009-bib-0103] Mitragotri, S. , P. A. Burke , and R. Langer . 2014. “Overcoming the Challenges in Administering Biopharmaceuticals: Formulation and Delivery Strategies.” Nature Reviews Drug Discovery 13, no. 9: 655–672. 10.1038/nrd4363. 25103255 PMC4455970

[wnan70009-bib-0104] Moeller, E. H. , and L. Jorgensen . 2008. “Alternative Routes of Administration for Systemic Delivery of Protein Pharmaceuticals.” Drug Discovery Today: Technologies 5, no. 2: e89–e94. 10.1016/j.ddtec.2008.11.005. 24981096

[wnan70009-bib-0105] Moffatt, K. , and R. F. Donnelly . 2021. “Chapter 17—Microneedle Technology.” In Drug Delivery Devices and Therapeutic Systems, edited by E. Chappel , 345–366. Academic Press. https://www.sciencedirect.com/science/article/pii/B9780128198384000043.

[wnan70009-bib-0106] Mohammed, Y. H. , M. Yamada , L. L. Lin , et al. 2014. “Microneedle Enhanced Delivery of Cosmeceutically Relevant Peptides in Human Skin.” PLoS One 9, no. 7: e101956. 10.1371/journal.pone.0101956. 25033398 PMC4102460

[wnan70009-bib-0107] Morganti, P. 2010. “Use and Potential of Nanotechnology in Cosmetic Dermatology.” Clinical, Cosmetic and Investigational Dermatology 3: 5–13. 10.2147/ccid.s4506. 21437055 PMC3047942

[wnan70009-bib-0108] Mortazavi, S. M. , F. Kobarfard , H. I. Maibach , and H. R. Moghimi . 2019. “Effect of Palmitic Acid Conjugation on Physicochemical Properties of Peptide KTTKS: A Preformulation Study.” Journal of Cosmetic Science 70, no. 6: 299–312. http://www.ncbi.nlm.nih.gov/pubmed/31829923. 31829923

[wnan70009-bib-0109] Nucara, L. , F. Greco , and V. Mattoli . 2015. “Electrically Responsive Photonic Crystals: A Review.” Journal of Materials Chemistry C 3, no. 33: 8449–8467. 10.1039/C5TC00773A.

[wnan70009-bib-0110] Ofokansi, K. C. , and M. U. Adikwu . 2007. “Formulation and Evaluation of Microspheres Based on Gelatin‐Mucin Admixtures for the Rectal Delivery of Cefuroxime Sodium.” Tropical Journal of Pharmaceutical Research 6, no. 4: 825–832. 10.4314/tjpr.v6i4.14666.

[wnan70009-bib-0111] Paniz, C. , D. Grotto , G. C. Schmitt , et al. 2005. “Fisiopatologia da deficiência de vitamina B12 e seu diagnóstico laboratorial.” Jornal Brasileiro de Patologia e Medicina Laboratorial 41, no. 5: 323–334. 10.1590/S1676-24442005000500007.

[wnan70009-bib-0112] Parhi, R. 2018. “16—Nanocomposite for Transdermal Drug Delivery.” In Applications of Nanocomposite Materials in Drug Delivery, edited by A. Inamuddin , M. Asiri , and A. Mohammad , 353–389. Woodhead Publishing. https://www.sciencedirect.com/science/article/pii/B9780128137413000169.

[wnan70009-bib-0113] Peppas, N. A. , P. Bures , W. Leobandung , and H. Ichikawa . 2000. “Hydrogels in Pharmaceutical Formulations.” European Journal of Pharmaceutics and Biopharmaceutics: Official Journal of Arbeitsgemeinschaft fur Pharmazeutische Verfahrenstechnik e.V 50, no. 1: 27–46. 10.1016/S0939-6411(00)00090-4. 10840191

[wnan70009-bib-0114] Peredo, A. P. , Y. K. Jo , G. Duan , G. R. Dodge , D. Lee , and R. L. Mauck . 2021. “Mechano‐Activated Biomolecule Release in Regenerating Load‐Bearing Tissue Microenvironments.” Biomaterials 265: 120255. 10.1016/j.biomaterials.2020.120255. 33099065 PMC7696653

[wnan70009-bib-0115] Petrusic, S. , and V. Koncar . 2016. “5—Controlled Release of Active Agents From Microcapsules Embedded in Textile Structures.” In Smart Textiles and Their Applications, edited by V. Koncar , 89–114. Woodhead Publishing. 10.1016/B978-0-08-100574-3.00005-9.

[wnan70009-bib-0116] Piacentini, E. , F. Bazzarelli , T. Poerio , et al. 2020. “Encapsulation of Water‐Soluble Drugs in Poly (Vinyl Alcohol) (PVA)‐ microparticles via Membrane Emulsification: Influence of Process and Formulation Parameters on Structural and Functional Properties.” Materials Today Communications 24: 100967. 10.1016/j.mtcomm.2020.100967.

[wnan70009-bib-0117] Purohit, T. J. , S. M. Hanning , and Z. Wu . 2018. “Advances in Rectal Drug Delivery Systems.” Pharmaceutical Development and Technology 23, no. 10: 942–952. 10.1080/10837450.2018.1484766. 29888992

[wnan70009-bib-0118] Ramakrishnan, Y. , N. M. Adzahan , Y. A. Yusof , and K. Muhammad . 2018. “Effect of Wall Materials on the Spray Drying Efficiency, Powder Properties and Stability of Bioactive Compounds in Tamarillo Juice Microencapsulation.” Powder Technology 328: 406–414. 10.1016/j.powtec.2017.12.018.

[wnan70009-bib-0119] Ramalho, M. J. , J. A. Loureiro , and M. C. Pereira . 2021. “Poly(Lactic‐Co‐Glycolic Acid) Nanoparticles for the Encapsulation and Gastrointestinal Release of Vitamin B9 and Vitamin B12.” ACS Applied Nano Materials 4, no. 7: 6881–6892. 10.1021/acsanm.1c00954.

[wnan70009-bib-0120] Raza, K. , M. Kumar , P. Kumar , et al. 2014. “Topical Delivery of Aceclofenac: Challenges and Promises of Novel Drug Delivery Systems.” BioMed Research International 2014: e406731. 10.1155/2014/406731. PMC4086417 25045671

[wnan70009-bib-0121] Ripoll, L. , and Y. Clement . 2016. “Polyamide Microparticles Containing Vitamin C by Interfacial Polymerization: An Approach by Design of Experimentation.” Cosmetics 3, no. 4: 38. 10.3390/cosmetics3040038.

[wnan70009-bib-0122] Roberts, M. S. , Y. Mohammed , M. N. Pastore , et al. 2017. “Topical and Cutaneous Delivery Using Nanosystems.” Journal of Controlled Release 247: 86–105. 10.1016/j.jconrel.2016.12.022. 28024914

[wnan70009-bib-0123] Ronnander, P. , L. Simon , H. Spilgies , A. Koch , and S. Scherr . 2018. “Dissolving Polyvinylpyrrolidone‐Based Microneedle Systems for In‐Vitro Delivery of Sumatriptan Succinate.” European Journal of Pharmaceutical Sciences 114: 84–92. 10.1016/j.ejps.2017.11.031. 29203152

[wnan70009-bib-0124] Runnsjö, A. , S. Liljedahl , D. Sagna , M. Ekblad , and J. Alenfall . 2022. “A Novel Microparticle Based Formulation for Topical Delivery of FOL‐005, a Small Peptide.” Journal of Pharmaceutical Sciences 111, no. 5: 1309–1317. 10.1016/j.xphs.2022.01.009. 35093338

[wnan70009-bib-0125] Santos, A. J. M. , S. Khemiri , S. Simões , C. Prista , I. Sousa , and A. Raymundo . 2023. “The Importance, Prevalence and Determination of Vitamins B6 and B12 in Food Matrices: A Review.” Food Chemistry 426: 136606. 10.1016/j.foodchem.2023.136606. 37356238

[wnan70009-bib-0126] Sharad, J. 2013. “Glycolic Acid Peel Therapy—A Current Review.” Clinical, Cosmetic and Investigational Dermatology 6: 281–288. 10.2147/CCID.S34029. 24399880 PMC3875240

[wnan70009-bib-0127] Shin, M. , H. K. Kim , and H. Lee . 2014. “Dopamine‐Loaded Poly(d,l‐Lactic‐Co‐Glycolic Acid) Microspheres: New Strategy for Encapsulating Small Hydrophilic Drugs With High Efficiency.” Biotechnology Progress 30, no. 1: 215–223. 10.1002/btpr.1835. 24281843

[wnan70009-bib-0128] Singh, P. , and A. Nanda . 2014. “Enhanced Sun Protection of Nano‐Sized Metal Oxide Particles Over Conventional Metal Oxide Particles: An In Vitro Comparative Study.” International Journal of Cosmetic Science 36, no. 3: 273–283. 10.1111/ics.12124. 24575878

[wnan70009-bib-0129] Singhvi, G. , P. Manchanda , N. Hans , S. K. Dubey , and G. Gupta . 2019. “Microsponge: An Emerging Drug Delivery Strategy.” Drug Development Research 80, no. 2: 200–208. 10.1002/ddr.21492. 30456763

[wnan70009-bib-0130] Smeets, A. , C. Clasen , and G. Van den Mooter . 2017. “Electrospraying of Polymer Solutions: Study of Formulation and Process Parameters.” European Journal of Pharmaceutics and Biopharmaceutics 119: 114–124. 10.1016/j.ejpb.2017.06.010. 28610878

[wnan70009-bib-0131] Sui, C. , Y. Lu , H.‐L. Gao , et al. 2013. “Synthesis of Mesoporous Calcium Phosphate Microspheres by Chemical Transformation Process: Their Stability and Encapsulation of Carboxymethyl Chitosan.” Crystal Growth & Design 13, no. 7: 3201–3207. 10.1021/cg400595s.

[wnan70009-bib-0132] Sui, C. , J. A. Preece , S.‐H. Yu , and Z. Zhang . 2018. “Novel Encapsulation of Water Soluble Inorganic or Organic Ingredients in Melamine Formaldehyde Microcapsules to Achieve Their Sustained Release in an Aqueous Environment.” RSC Advances 8, no. 52: 29495–29498. 10.1039/c8ra05533e. 35547310 PMC9085263

[wnan70009-bib-0133] Sui, C. , J. A. Preece , Z. Zhang , and S.‐H. Yu . 2021. “Efficient Encapsulation of Water Soluble Inorganic and Organic Actives in Melamine Formaldehyde Based Microcapsules for Control Release Into an Aqueous Environment.” Chemical Engineering Science 229: 116103. 10.1016/j.ces.2020.116103.

[wnan70009-bib-0134] Takahashi, M. , Y. Taguchi , and M. Tanaka . 2010. “Microencapsulation of Hydrophilic Solid Powder as a Flame Retardant With Epoxy Resin by Using Interfacial Reaction Method.” Polymers for Advanced Technologies 21, no. 3: 224–228. 10.1002/pat.1410.

[wnan70009-bib-0135] Teutonico, D. , and G. Ponchel . 2011. “Patches for Improving Gastrointestinal Absorption: An Overview.” Drug Discovery Today 16, no. 21–22: 991–997. 10.1016/j.drudis.2011.05.013. 21683153

[wnan70009-bib-0136] Trindade, M. A. , and C. R. Grosso . 2000. “The Stability of Ascorbic Acid Microencapsulated in Granules of Rice Starch and in Gum Arabic.” Journal of Microencapsulation 17, no. 2: 169–176. 10.1080/026520400288409. 10738692

[wnan70009-bib-0137] Tulini, F. L. , V. B. Souza , M. Thomazini , et al. 2017. “Evaluation of the Release Profile, Stability and Antioxidant Activity of a Proanthocyanidin‐Rich Cinnamon ( *Cinnamomum zeylanicum* ) Extract Co‐Encapsulated With α‐Tocopherol by Spray Chilling.” Food Research International 95: 117–124. 10.1016/j.foodres.2017.03.010. 28395819

[wnan70009-bib-0138] Waghulde, M. R. , and J. B. Naik . 2016. “Poly‐e‐Caprolactone‐Loaded Miglitol Microspheres for the Treatment of Type‐2 Diabetes Mellitus Using the Response Surface Methodology.” Journal of Taibah University Medical Sciences 11, no. 4: 364–373. 10.1016/j.jtumed.2016.03.006.

[wnan70009-bib-0139] Wang, R. , C. Ma , H. Yan , et al. 2023. “Preparation and Characterization of GX‐50 and Vitamin C Co‐Encapsulated Microcapsules by a Water‐in‐Oil‐in‐Water (W 1/O/W 2) Double Emulsion–Complex Coacervation Method.” Langmuir 39, no. 39: 13863–13875. 10.1021/acs.langmuir.3c01360. 37733306

[wnan70009-bib-0140] Wang, S. , H. Liu , D. Wu , and X. Wang . 2021. “Temperature and pH Dual‐Stimuli‐Responsive Phase‐Change Microcapsules for Multipurpose Applications in Smart Drug Delivery.” Journal of Colloid and Interface Science 583: 470–486. 10.1016/j.jcis.2020.09.073. 33011414

[wnan70009-bib-0141] Wang, Y. , H. Tao , K. Wang , et al. 2021. “Nanocapsules Formed by Interactions Between Chondroitin Sulfate and Egg White Protein for Encapsulating Hydrophilic Ingredients.” Green Chemistry 23, no. 19: 7566–7575. 10.1039/D1GC01706C.

[wnan70009-bib-0142] Windbergs, M. , Y. Zhao , J. Heyman , and D. A. Weitz . 2013. “Biodegradable Core–Shell Carriers for Simultaneous Encapsulation of Synergistic Actives.” Journal of the American Chemical Society 135, no. 21: 7933–7937. 10.1021/ja401422r. 23631388

[wnan70009-bib-0143] Wu, P.‐C. , Y.‐B. Huang , J.‐S. Chang , M.‐J. Tsai , and Y.‐H. Tsai . 2003a. “Design and Evaluation of Sustained Release Microspheres of Potassium Chloride Prepared by Eudragit.” European Journal of Pharmaceutical Sciences 19, no. 2: 115–122. 10.1016/S0928-0987(03)00069-1. 12791413

[wnan70009-bib-0144] Wu, P.‐C. , Y.‐B. Huang , J.‐S. Chang , M.‐J. Tsai , and Y.‐H. Tsai . 2003b. “Preparation and Evaluation of Sustained Release Microspheres of Potassium Chloride Prepared With Ethylcellulose.” International Journal of Pharmaceutics 260, no. 1: 115–121. 10.1016/s0378-5173(03)00255-2. 12818816

[wnan70009-bib-0145] Xia, C. , W. Wang , L. Wang , H. Liu , and J. Xiao . 2019. “Multilayer Zein/Gelatin Films With Tunable Water Barrier Property and Prolonged Antioxidant Activity.” Food Packaging and Shelf Life 19: 76–85. 10.1016/j.fpsl.2018.12.004.

[wnan70009-bib-0146] Xu, J. , M. Tam , S. Samaei , et al. 2017. “Mucoadhesive Chitosan Hydrogels as Rectal Drug Delivery Vessels to Treat Ulcerative Colitis.” Acta Biomaterialia 48: 247–257. 10.1016/j.actbio.2016.10.026. 27769943

[wnan70009-bib-0147] Yamazoe, H. , C. Kominami , and H. Abe . 2022. “Superior Adhesion of a Multifunctional Protein‐Based Micropatch to Intestinal Tissue by Harnessing the Hydrophobic Effect.” Small Methods 6, no. 6: 2200153. 10.1002/smtd.202200153. 35460203

[wnan70009-bib-0148] Yang, Y. , Y. Chen , Z. Hou , et al. 2020. “Responsive Photonic Crystal Microcapsules of Block Copolymers With Enhanced Monochromaticity.” ACS Nano 14, no. 11: 16057–16064. 10.1021/acsnano.0c07898. 33191731

[wnan70009-bib-0149] Yeom, C. K. , S. B. Oh , J. W. Rhim , and J. M. Lee . 2000. “Microencapsulation of Water‐Soluble Herbicide by Interfacial Reaction. I. Characterization of Microencapsulation.” Journal of Applied Polymer Science 78, no. 9: 1645–1655. 10.1002/1097-4628(20001128)78:9<1645::AID-APP100>3.0.CO;2-2.

[wnan70009-bib-0150] Yuan, Y. , M. Yin , Q. Zhai , and M. Chen . 2024. “The Encapsulation Strategy to Improve the Survival of Probiotics for Food Application: From Rough Multicellular to Single‐Cell Surface Engineering and Microbial Mediation.” Critical Reviews in Food Science and Nutrition 64, no. 10: 1–17. 10.1080/10408398.2022.2126818. 36168909

[wnan70009-bib-0151] Zaki Rizkalla, C. M. , R. latif Aziz , and I. I. Soliman . 2011. “In Vitro and In Vivo Evaluation of Hydroxyzine Hydrochloride Microsponges for Topical Delivery.” AAPS PharmSciTech 12, no. 3: 989–1001. 10.1208/s12249-011-9663-5. 21800216 PMC3167254

[wnan70009-bib-0152] Zhang, Z. , R. Saunders , and C. R. Thomas . 1999. “Mechanical Strength of Single Microcapsules Determined by a Novel Micromanipulation Technique.” Journal of Microencapsulation 16, no. 1: 117–124. 10.1080/026520499289365. 9972508

[wnan70009-bib-0153] Zhao, Q. , J. Liu , W. Zhu , et al. 2015. “Dual‐Stimuli Responsive Hyaluronic Acid‐Conjugated Mesoporous Silica for Targeted Delivery to CD44‐Overexpressing Cancer Cells.” Acta Biomaterialia 23: 147–156. 10.1016/j.actbio.2015.05.010. 25985912

[wnan70009-bib-0154] Zhao, Y. , Z. Luo , M. Li , et al. 2015. “A Preloaded Amorphous Calcium Carbonate/Doxorubicin@Silica Nanoreactor for pH‐Responsive Delivery of an Anticancer Drug.” Angewandte Chemie International Edition 54, no. 3: 919–922. 10.1002/anie.201408510. 25422068

